# Verteporfin-induced proteotoxicity impairs cell homeostasis and survival in neuroblastoma subtypes independent of YAP/TAZ expression

**DOI:** 10.1038/s41598-023-29796-2

**Published:** 2023-03-07

**Authors:** Alexandra-Larisa Condurat, Sepideh Aminzadeh-Gohari, Mirjana Malnar, Nicole Schider, Leonie Opitz, Ria Thomas, Vishal Menon, Barbara Kofler, Jan Pruszak

**Affiliations:** 1 Emmy Noether-Group for Stem Cell Biology, Department of Molecular Embryology, Institute of Anatomy and Cell Biology, Faculty of Medicine, University of Freiburg, Freiburg, Germany; 2 Spemann Graduate School of Biology and Medicine and Faculty of Biology, University of Freiburg, Freiburg, Germany; 3 Research Program for Receptor Biochemistry and Tumor Metabolism, Department of Pediatrics, University Hospital of the Paracelsus Medical University, Salzburg, Austria; 4 Institute of Anatomy and Cell Biology, Paracelsus Medical University, Salzburg, Austria; 5 Center for Anatomy and Cell Biology, Salzburg and Nuremberg, Paracelsus Medical University, Salzburg, Austria

**Keywords:** Targeted therapies, Cancer therapy, Paediatric cancer, Tumour heterogeneity

## Abstract

Neuroblastoma (NB) is a highly aggressive extracranial solid tumor in children. Due to its heterogeneity, NB remains a therapeutic challenge. Several oncogenic factors, including the Hippo effectors YAP/TAZ, are associated with NB tumorigenesis. Verteporfin (VPF) is an FDA-approved drug shown to directly inhibit YAP/TAZ activity. Our study aimed to investigate VPF’s potential as a therapeutic agent in NB. We show that VPF selectively and efficiently impairs the viability of YAP/TAZ-expressing NB GI-ME-N and SK-N-AS cells, but not of non-malignant fibroblasts. To investigate whether VPF-mediated NB cell killing is YAP-dependent, we tested VPF potency in CRISPR-mediated YAP/TAZ knock-out GI-ME-N cells, and BE(2)-M17 NB cells (a *MYCN-*amplified, predominantly YAP-negative NB subtype). Our data shows that VPF-mediated NB cell killing is not dependent on YAP expression. Moreover, we determined that the formation of higher molecular weight (HMW) complexes is an early and shared VPF-induced cytotoxic mechanism in both YAP-positive and YAP-negative NB models. The accumulation of HMW complexes, involving STAT3, GM130 and COX IV proteins, impaired cell homeostasis and triggered cell stress and cell death mechanisms. Altogether, our study shows significant in vitro and in vivo VPF-induced suppression of NB growth, making VPF a potential therapeutic candidate against NB.

## Introduction

Neuroblastoma (NB) is a pediatric tumor with embryonic origins that arises from neural crest precursors of the sympathoadrenal lineage^1,2^. This tumor type is most frequently detected during infancy and early childhood, with 90% of diagnosed patients being < 5 years old^3^. Clinical outcome in NB patients depends on age at the time of diagnosis, the mutational profile, differentiation status and metastatic spread of the tumor^3,4^. Despite the use of multimodal treatment strategies including surgery, chemo-/radiotherapy, immunotherapy, stem cell transplantation and differentiation agents, the overall survival of high-risk NB patients remains low^3,5,6^.

The established oncogenic markers C-MYC and N-MYC are predictors of poor outcome in distinct NB high-risk subtypes^4,7–9^. Typically, C-MYC is overexpressed and/or active in stem-like undifferentiated NBs, while N-MYC activity is predominant in poorly differentiated adrenergic NB subtypes^7,9–11^. Despite intense efforts, transcription factors, including the MYC family members, are generally considered undruggable targets due to their localization, complex mechanisms of action involving multiprotein complexes, and role in non-malignant cells^12^. Therefore, considerable efforts are dedicated to the identification of additional molecular vulnerabilities and therapeutic agents that would be effective in targeting and suppressing NB cells.

Recent studies have described the contribution of the Hippo tumor suppressor pathway downstream effectors YAP (Yes-associated protein) and its paralog TAZ (transcriptional co-activator with PDZ-binding motif; WWTR1) in NB tumorigenesis, treatment resistance and relapse, highlighting the functional relevance of targeting YAP/TAZ in NB tumors^13–22^. YAP/TAZ are transcriptional co-activators that play a central role in stem cell modulation and tissue regeneration^23–25^. Their oncogenic roles are linked to promoting cell survival, proliferation, migration, and drug resistance, in part via their association with various transcription factors, including the TEA domain (TEAD) transcription factors^22,24,25^. Verteporfin (VPF), an FDA-approved drug clinically used as a photosensitizer in photodynamic therapy, was shown to directly bind to YAP and prevent its binding to TEAD factors, inhibiting its transcriptional activity^26–29^. Therefore, our study aimed to investigate the repurposing potential of VPF as a therapeutic agent in NB subtypes.

We tested VPF potency across distinctive NB subtypes, more specifically in *MYCN*-non-amplified (*MYCN-NA)* and predominantly YAP-positive, as well as in *MYCN*-amplified (*MYCN-A)* and predominantly YAP-negative NB cell lines. Our analysis shows that VPF efficiently and selectively impaired cell viability in YAP/TAZ-expressing NB cells, but not in YAP-expressing non-malignant fetal fibroblasts. To address whether VPF potency in NB is strictly dependent on YAP, we tested VPF cytotoxicity in CRISPR/Cas9-mediated YAP/TAZ knock-out lines, as well as the predominantly YAP/TAZ-negative BE(2)-M17 NB cell line. Collectively, these experiments strongly demonstrate that VPF-induced NB cell death is not dependent on YAP/TAZ expression, suggesting additional molecular mechanisms as mediators and a broader therapeutic potential of VPF in NB. Among the molecular effects of VPF treatment we detected the downregulation of oncogenic factors, including YAP, TAZ, MYC, N-MYC, of cell cycle and cell migration effectors, and functional impairment of cell migration and survival. Moreover, we determined that in both *MYCN-A* and *MYCN-NA* NB subtypes, VPF cytotoxicity is in part mediated via the formation and accumulation of oligomeric protein complexes, involving STAT3, GM130 and COX IV proteins. Altogether, we show that VPF is a potent in vitro and in vivo suppressor of NB growth, independent of YAP/TAZ expression.

## Results

### YAP/TAZ-expressing neuroblastoma cells co-express early neural crest markers

**Figure 1:**
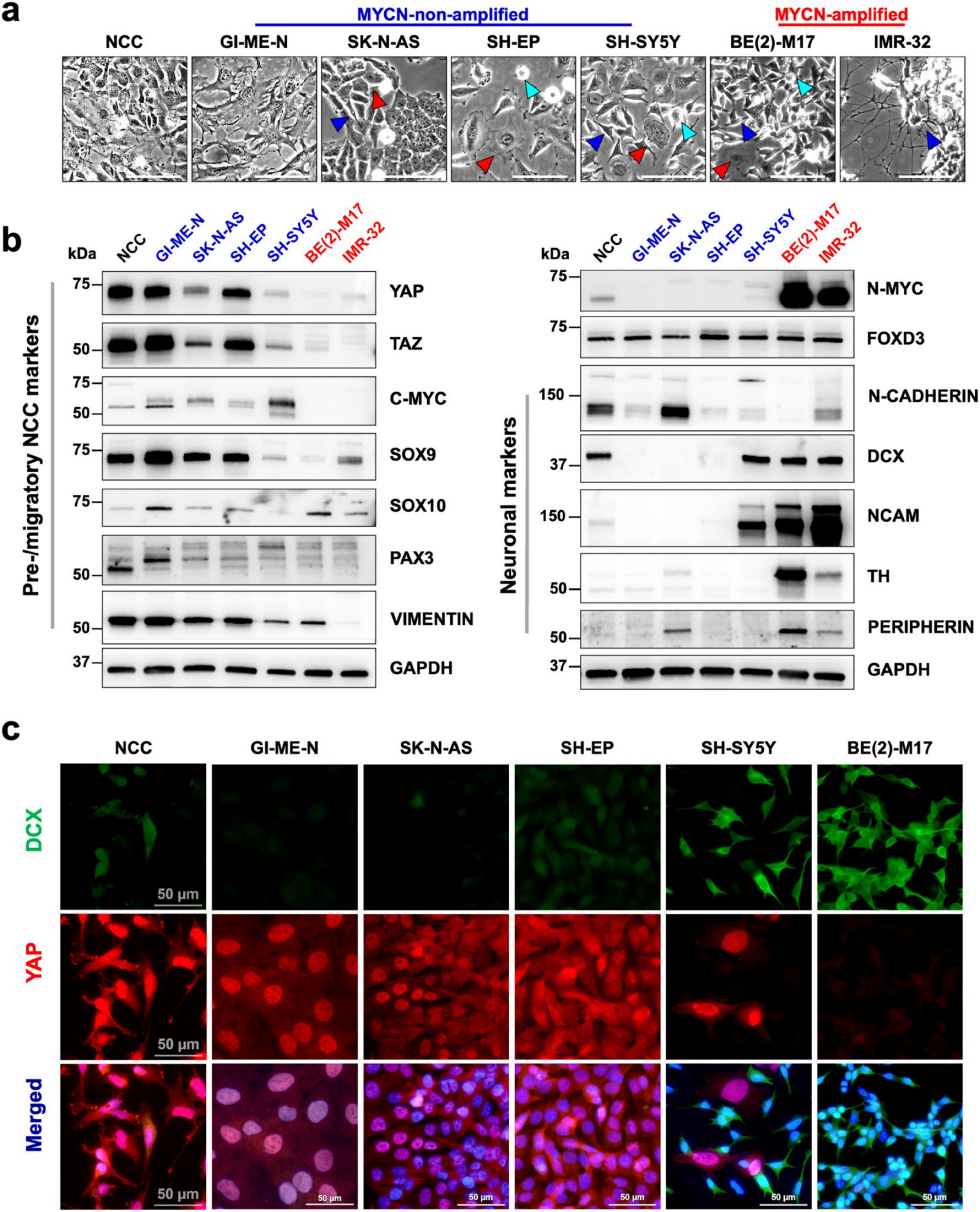
YAP/TAZ are highly expressed in neuroblastoma models that phenotypically and molecularly resemble early neural crest cells. (**a**) Phase-contrast images of hESC-derived neural crest cells (NCC), *MYCN*-non-amplified (GI-ME-N, SK-N-AS, SH-EP, SH-SY5Y) and *MYCN*-amplified (BE(2)-M17, IMR-32) metastatic neuroblastoma cell lines; scale bars: 100 µm. (**b**) Comparative western blot analysis of YAP/TAZ co-expression with neuroblastoma biomarkers (N-MYC, C-MYC), pre-/migratory NCC markers (SOX9/10, PAX3, FOXD3) and neuronal markers (N-CADHERIN, DCX, NCAM, TH, PERIPHERIN) showing a strong overlap of YAP/TAZ expression with early NCC markers. (**c**) Immunofluorescence analysis of DCX (green) and YAP (red) co-expression in NCC and NB cell lines showing the absence/low expression of YAP in DCX + cells; scale bars: 50 µm. Arrowheads: blue = neuroblast/neuronal-like cell, red = large/mesenchymal-like cell, turquoise = round /low adherent cell.

We investigated the expression pattern of the Hippo effectors YAP/TAZ in both *MYCN*-A and *MYCN*-NA NB models. The NB cell lines tested are metastasis-derived, commonly used in NB research and with a well characterized tumorigenic potential in animal models (Suppl. Table 1). Morphologically, the *MYCN*-NA NB lines GI-ME-N, SK-N-AS and SH-EP lines are predominantly mesenchymal-like (red arrow), similar to hES-derived neural crest cells (NCC), with only a small fraction of neuroblast-like (blue arrowheads) and low-adherent (turquoise arrowheads) cells present (Fig. 1a). On the contrary, the *MYCN*-A NB lines BE(2)-M17 and IMR-32 show a predominant neuroblast/neuronal-like morphology (Fig. 1a). Notably, the SH-SY5Y NB line, known for its heterogeneity^24^, presents a higher morphological diversity of NB cellular subtypes: mesenchymal, intermediate/low-adherent and neuroblast-like cell subpopulations. At protein level, YAP/TAZ are highly expressed in NCCs and *MYCN*-NA NB cells lines, but barely detectable in *MYCN*-A NB lines (Fig. 1b). Moreover, YAP/TAZ expression was found to overlap with C-MYC and other early neural-crest markers (SOX9—SRY-Box Transcription Factor 9, PAX3—Paired Box 3, vimentin), but not neuronal markers, such as DCX—doublecortin, NCAM—Neural Cell Adhesion Molecule, TH—Tyrosine Hydroxylase or peripherin. On the other hand, N-MYC expression is barely detectable in the *MYCN*-NA/YAP-expressing NB lines, but very pronounced in *MYCN-*A NB lines and generally overlaps with early (DCX, NCAM) and mature neuronal (TH, peripherin) markers. FOXD3 (Forkhead Box D3)—an early neural crest specifier, as well as a late neural crest cell fate modulator^30^, is detected in both *MYCN*-NA and *MYCN*-A NB lines. The heterogeneity of the SH-SY5Y cell line is also noticeable at molecular level, where we detected the expression of both early NCC markers as well as neuronal markers (Fig. 1b), in line with previously reported data^24,31,32^. Indirect immunofluorescence analyses of YAP and DCX expression (Fig. 1c) illustrate at single cell resolution that generally their expression is mutually exclusive, with early NCCs maintaining a transient overlap following neuroepithelial fate transition. Overall, a strong YAP signal is observed in NCCs and *MYCN*-NA NB cells, but barely detected in *MYCN-*A BE(2)M-17 NB cells, which are predominantly DCX positive. To test whether the in vitro pattern of YAP/TAZ expression is also observed in patient samples, we used the cBioPortal database and examined *YAP/WWTR1* expression in 143 NB samples included in the Neuroblastoma TARGET dataset^4,33,34^. Indeed, we confirmed that also in NB patient samples *YAP1/WWTR1* expression positively correlates with *MYC* expression, but negatively correlates with *MYCN* and *DCX* expression. In contrast, *DCX* expression positively correlates with *MYCN* expression, but negatively correlates with *MYC* expression (Suppl. Figure 1). Of note, some patient samples appear to indicate both *MYC* and *MYCN* expression, which might be indicative of heterogeneous tumors with mixed and co-existing subpopulations of phenotypically and functionally divergent NB cells.

### Neuroblastoma cells are more susceptible to VPF-induced cell death compared to non-malignant fetal fibroblasts

**Figure 2:**
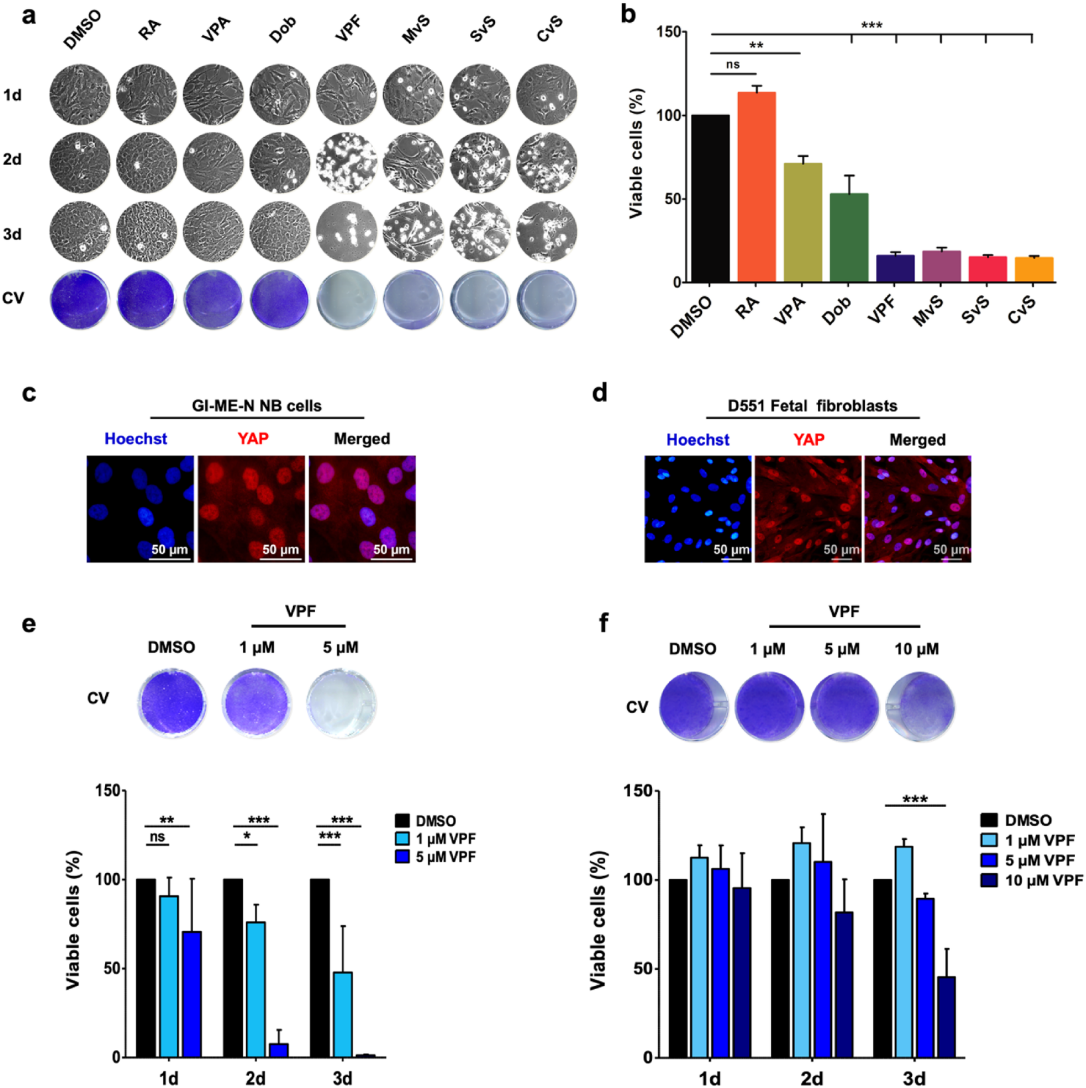
Verteporfin is a potent and selective therapeutic agent in GI-ME-N YAP-expressing neuroblastoma cells. (**a**) Phase-contrast images of GI-ME-N cells treated for 3 consecutive days with various compounds (n = 4; DMSO = vehicle control; RA = retinoic acid, 10 µM; VPA = valproic acid, 2 mM; Dob = dobutamine, 20 µM; VPF = verteporfin, 10 µM; MvS = mevastatin, 10 µM; SvS = simvastatin, 10 µM; CvS = cerivastatin, 10 µM; CV = crystal violet viability staining at day 3 post-treatment). (**b**) Corresponding quantification of viable GI-ME-N cells by crystal violet staining following the 3 days treatment. Bars represent average ± SEM (n = 4; normalized against DMSO controls set to 100%), statistical significance was determined using a one-way analysis of variance (ANOVA) with Dunnett’s *post-hoc* test: ns = not statistically significant; **p ≤ 0.01; ***p ≤ 0.001. Indirect immunofluorescence of YAP expression in GI-ME-N NB cells (**c**) and non-malignant fetal fibroblasts—D551 cells (**d**), highlighting a strong YAP nuclear localization in both cell systems; scale bars: 50 µm. Cell viability assessed by crystal violet (CV) staining of 3 days-treated GI-ME-N cells (**e**) and D551 fibroblasts (**f**) with increased concentrations of VPF and the corresponding quantification. DMSO treatment was used as a vehicle control. Bars represent average ± SEM (n ≥ 4; normalized against DMSO controls set to 100%), statistical significance was determined using a two-way analysis of variance (ANOVA) followed by Bonferroni’s *post-hoc* test: ns = not statistically significant; *p ≤ 0.05; **p ≤ 0.01; ***p ≤ 0.001.

We used the GI-ME-N NB cell line as a representative YAP/TAZ-expressing NB model to test VPF potency—an effective YAP/TAZ inhibitor^28^, in inducing cell death compared to NB therapeutic agents (retinoic acid—RA, valproic acid—VPA), and other reported blockers of YAP/TAZ activity (dobutamine—Dob, mevastatin—MvS, simvastatin—SvS, cerivastatin—CvS)^3,5,25,28^. We treated GI-ME-N cells for 3 days with the selected compounds and used crystal violet assay to assess impairment in viability. VPF and statins were most effective in inducing cell death in GI-ME-N cells (< 25% viable cells), compared to Dob (> 50% viable cells) and VPA (> 70% viable cells) (Fig. 2a,b). As previously reported^24^, we noticed that RA treatment in YAP-expressing NB cells does not have suppressive or cytotoxic effects (Fig. 2a,b). To evaluate the tumor-selective potential of VPF, we compared its cytotoxicity in GI-ME-N NB cells and non-malignant D551 fetal fibroblasts, both cell models highly expressing YAP (Fig. 2c,d). We show that following a 3 days treatment with 10 µM VPF > 40% D551 fibroblasts were viable (Fig. 2f), while at the same VPF concentration < 20% GI-ME-N cells remained viable (Fig. 2a,b). A more notable tumor-selective effect was detected at lower VPF concentrations, wherein the 3 days treatment with 5 µM VPF led to < 10% viable GI-ME-N cells, while > 85% viable D551 cells were detected under the same treatment conditions (Fig. 2e,f). To show that VPF potency was not limited to the GI-ME-N NB cell model, we performed similar experiments in another YAP/TAZ-highly expressing NB cell line—the SK-N-AS cell line (also see Fig. 1). Indeed, treatment of SK-N-AS cells with 5 µM VPF significantly decreased cell viability over time, with < 40% viable cells at 48 h and < 10% viable cells at 72 h post-treatment (p.t.) (Supp. Figure 2). Overall, these experiments demonstrate that VPF induces a pronounced viability impairment in NB cells, in a dose and time dependent manner, while being less toxic in non-malignant YAP-expressing fibroblasts.

### VPF treatment leads to the formation of higher-molecular weight complexes and impairs cell homeostasis, migration and survival in NB cells

**Figure 3:**
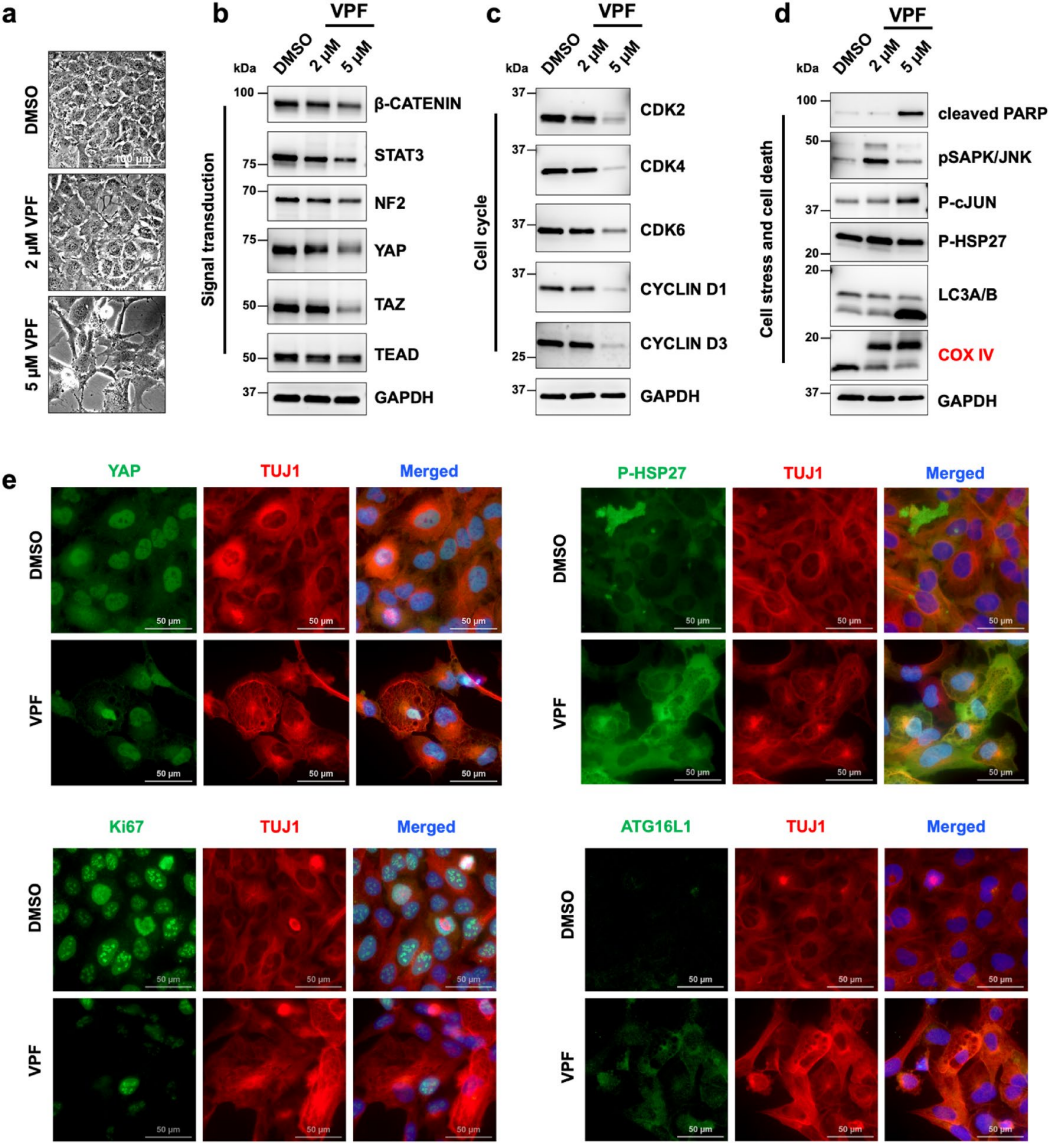
Verteporfin downregulates the expression of oncogenic, pro-survival and cell-cycle modulators and drives the activation of cell stress and cell death effectors. (**a**) Representative phase-contrast images of GI-ME-N cells treated for 24 h with DMSO, 2 µM or 5 µM VPF; scale bar: 100 µm. Western blot analysis of GI-ME-N cells treated for 24 h with either 2 or 5 µM VPF shows the downregulation of oncogenic and pro-survival mediators (**b**), downregulation of cell cycle modulators (**c**) and activation of cell stress and cell death mediators (**d**), compared to the DMSO control. Moreover, VPF-treated GI-ME-N cells indicate the formation of COX IV (red) higher molecular weight complexes. (**e**) Immunofluorescence analysis of GI-ME-N cells treated for 48 h with 5 µM VPF illustrate a reduction of YAP expression and Ki67-positive cells, as well as the activation of cell-stress mediators phospho-Ser82-HSP27 and ATG16L1; scale bars: 50 µm**.**

The anti-tumor effects of VPF have been attributed to various molecular mechanisms, including direct inhibition of YAP-TEAD binding, direct inhibition of kinases and formation of proteotoxic high molecular weight complexes^35,36^. Therefore, we used various molecular and functional assays to identify the mechanisms of VPF-mediated cytotoxicity in NB cells. We observed that 24 h treatment of GI-ME-N cells with VPF was sufficient to induce noticeable cytotoxic effects, in a dose-dependent manner (Fig. 3a). By performing a phospho-kinase array of GI-ME-N cells treated for 24 h with 5 µM VPF, we identified changes in the phosphorylation profile of several major signaling cascades and cellular processes (Supp. Figure 3a,b). We detected increased phosphorylation of cell stress modulators (p38, HSP27) and reduced phosphorylation of cell homeostasis regulators (WNK1, HSP60), transcription factors (STAT protein family members), cell metabolism and protein synthesis (p70 S6 kinase), cell survival (AKT, ERK) and cell cycle (p27) regulators. The array observations were confirmed by western blotting, which indicated besides an evident downregulation of central oncogenic factors (YAP/TAZ, β-catenin and STAT3; Fig. 3b), the downregulation of cell-cycle effectors—CDK2/4/6 and cyclin D1/3 (Fig. 3c), and upregulation of cell stress and cell death mediators—P–c-Jun, pSAPK/JNK and cleaved PARP (Fig. 3d). Indirect immunofluorescent images of GI-ME-N cells at 48 h post 5 µM VPF treatment reinforced the western blot findings, illustrating a decrease in YAP immunodetection, a decrease in proliferative/Ki67 positive cells, and activation of cell stress mechanisms, as indicated by enhanced P-HSP27 and ATG16L immunostaining (Fig. 3e). The phospho-kinase array also revealed that VPF treatment caused a decreased phosphorylation of cell migration effectors SRC and PLC-γ1 (Supp. Figure 3a,b). To address the functional consequences of VPF treatment on cell migration, we examined additional cell motility modulators by western blotting and performed two distinct in vitro migration assays. At protein level, we found that VPF treatment led to the downregulation of several pro-migratory mediators: total FAK, β1-integrin, CDC42 and RAC1/2/3 expression (Supp. Figure 4a), which functionally correlated with a significant impairment in cell migration, as demonstrated by migration scratch assays (Supp. Figure 4b) and transwell migration assays (Supp. Figure 4c,d). The observed reduction in migratory potential was not due to increased cell death, as illustrated by cells remaining in the upper transwell compartment (Supp. Figure 4e). Notably, western blot analysis revealed a pronounced higher molecular weight (HMW) band following COX IV immunodetection in the VPF-treated samples only, which was in keeping with previous reports wherein the formation of proteotoxic HMW oligomers were seen as a VPF-induced tumor-selective mechanism^37^.

**Figure 4:**
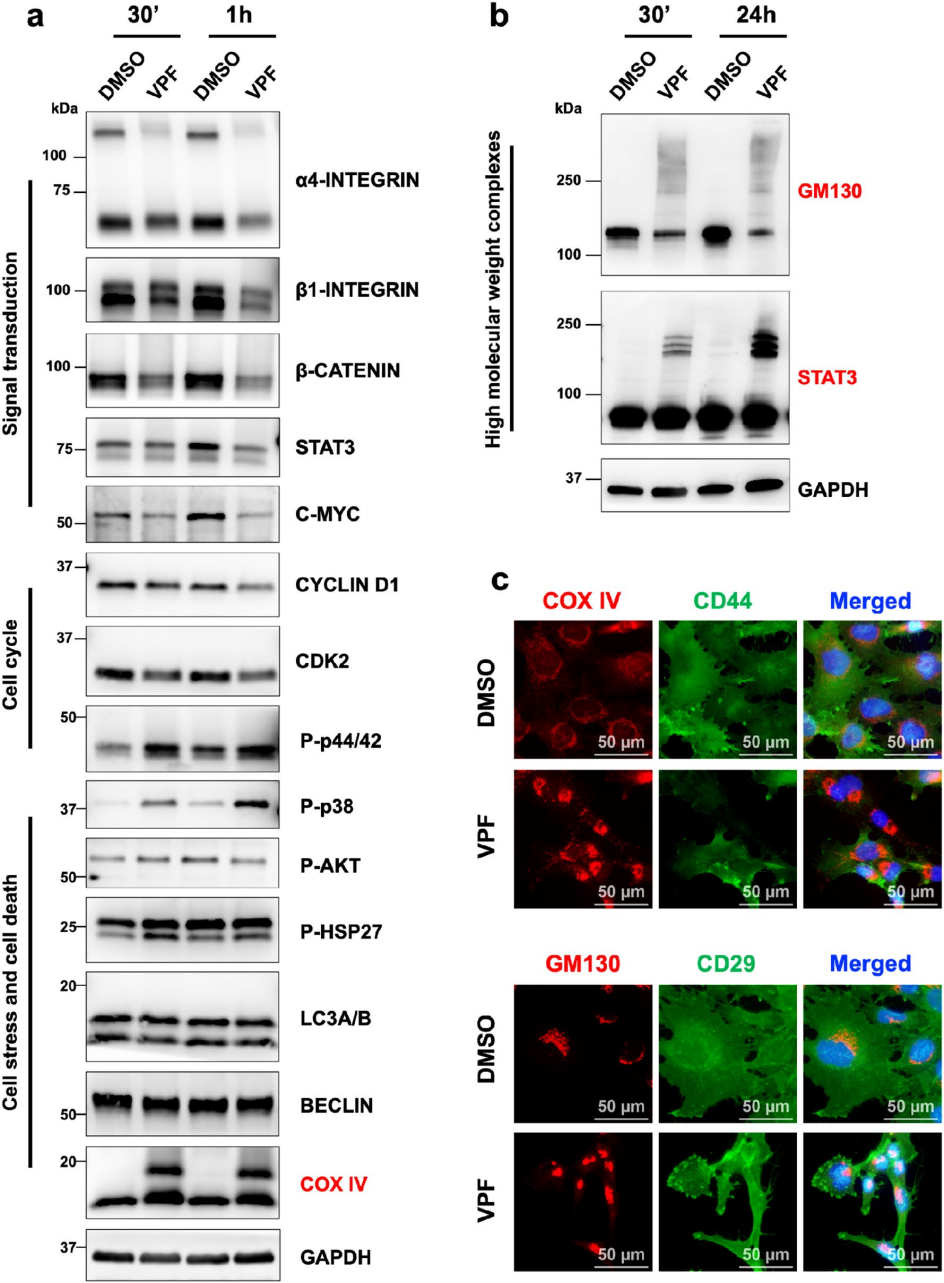
Short verteporfin treatment is sufficient to disrupt cell homeostasis via the formation of proteotoxic higher molecular weight complexes. (**a**) Western blot analysis of GI-ME-N cells treated for 30 min (30’) or 1 h with 5 µM VPF shows marked downregulation of pro-survival and migratory molecules (α4-/β1-integrin subunits), transcription modulators (STAT3, β-CATENIN, C-MYC), cell cycle factors (CYCLIN D1, CDK2), and activation of cell stress mechanisms (activating phosphorylation of p38, HSP27), and formation of COX IV higher molecular weight complexes—HMW (red). (**b**) Western blot analysis of GI-ME-N cells treated for 30’or 24 h with 5 µM VPF shows the formation and accumulation of GM130 and STAT3 HMW. (**c**) Immunofluorescence images of GI-ME-N cells treated for 24 h with 5 µM VPF highlighting the altered subcellular appearance of COX IV and GM130, respectively; scale bars: 50 µm. CD44 and CD29 surface antigen immunodetection was used as cell membrane counterstaining.

To confirm the formation of HMW complexes as an early VPF-induced cytotoxic mechanism, we treated GI-ME-N cells with 5 µM VPF for 30 min or 1 h and performed immunoblot analysis (Fig. 4a). As early as 30 min post VPF treatment, we detected pronounced COX IV HMW, and consecutively activated cell stress signaling (P-p38, P-HSP27, P-p44/42), as well as reduced pro-proliferative and pro-survival mediators (α4-integrin, β1-integrin, β-catenin, STAT3, C-MYC). Moreover, we studied other proteins previously reported to form proteotoxic oligomeric complexes following VPF treatment^37^, and were able to also attest the formation and accumulation of STAT3 and GM130 HMW oligomers (Fig. 4b). To further confirm the presence of these complexes, we performed immunofluorescence staining of GI-ME-N cells treated for 24 h with 5 µM VPF and show the formation of COX IV and GM130 aggregates (Fig. 4c). Altogether, our data indicates that VPF treatment induces a rapid formation of cytotoxic HMW complexes, and that their accumulation may cause an imbalance in cellular homeostasis and, consequently, activation of cell stress and eventual impairment of cell proliferation, migration and survival.

### YAP/TAZ expression is not required for VPF-induced cell death in neuroblastoma cells

**Figure 5:**
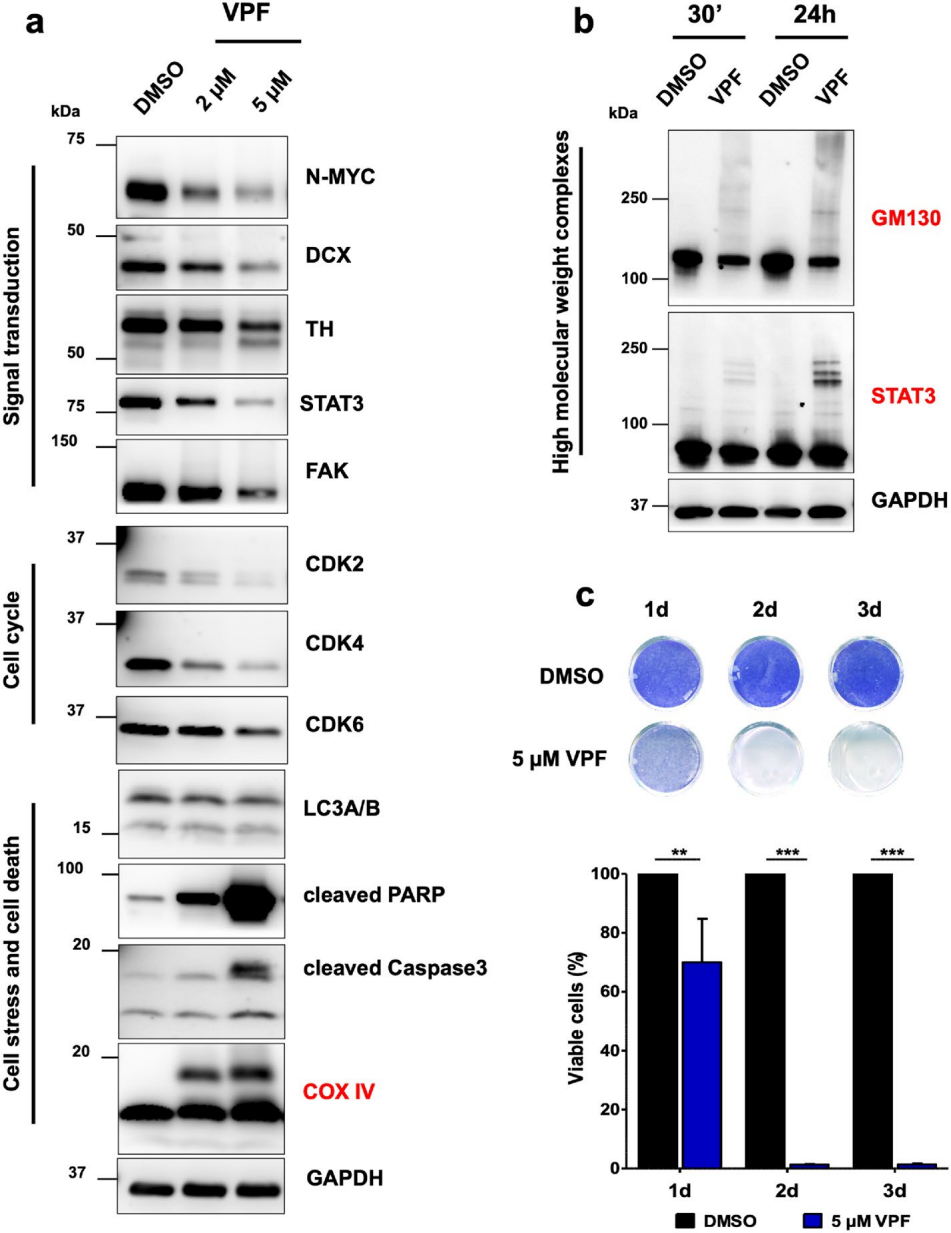
Verteporfin efficiently targets BE(2)-M17 cells, a predominantly YAP-negative and *MYCN*-amplified NB subtype. (**a**) Western blot analysis of BE(2)-M17 cells treated for 24 h with either 2 or 5 µM VPF, showing the downregulation of transcriptional regulators (N-MYC, STAT3) and cell cycle progression factors (CDK2/4/6), the upregulation of cell death regulators (cleaved PARP, cleaved caspase-3) and the formation of COX IV HMW complexes (red). (**b**) Western blot analysis of BE(2)-M17 cells treated for 30 min (30’) or 24 h with 5 µM VPF shows the formation and accumulation of GM130 and STAT3 high-molecular weight protein complexes. (**c**) Representative images of viable BE(2)-M17 cells after 1 to 3 days of 5 µM VPF treatment, as assessed by crystal violet staining, and the corresponding quantification graph. Data is represented as average ± SEM (n = 5; normalized against DMSO controls set to 100%). Two-way analysis of variance (ANOVA) followed by Bonferroni *post-hoc* tests was used to assess statistical significance (**p ≤ 0.01; ***p ≤ 0.001).

VPF oncosuppressive effects can be mediated through various mechanism, both dependent on as well as independent of light activation or YAP expression^36–46^. Thus, we aimed to investigate whether YAP/TAZ expression is required for VPF-induced NB cell death. For this purpose, we used CRISPR/Cas9-mediated genome editing and generated YAP and TAZ GI-ME-N knock-out (KO) lines and tested their sensitivity to VPF-induced cell death. The control (CTRL) KO line was generated using a non-targeting 20 nt scramble guide RNA. Protein immunoblotting confirmed the on-target knock-out efficacy of YAP and TAZ (Supp. Figure 5a). We then treated the parental, control, YAP and TAZ KO lines for three consecutive days with increasing concentrations of VPF (1 µM-10 µM) and included compounds that showed no effect in parental GI-ME-N cells (RA and VPA). As illustrated in Supp. Figure 5b and 5c, VPF induced significant cell-killing effects not only in the parental and KO control lines, but also in the YAP or TAZ GI-ME-N KO lines, providing first evidence to suggest that neither YAP nor TAZ expression is necessary for VPF-induced NB cell death. To further validate this observation, we next tested VPF potency in BE(2)-M17 cells, a *MYCN-*A NB line that is predominantly YAP/TAZ-negative (as illustrated in Fig. 1b,c). This approach was used to both validate the YAP/TAZ-independent effects of VPF on cell viability and to test whether VPF can be used to target diverse NB subtypes, including *MYCN-*A lines. Thus, we treated BE(2)-M17 cells with 2 and 5 µM VPF for 24 h and detected the downregulation of oncogenic effectors (N-MYC, DCX, STAT3, FAK), of cell-cycle mediators (CDK2/4/6) and activation of cell death effectors (cleaved PARP, cleaved Caspase3) (Fig. 5a). Moreover, similarly to GI-ME-N data, VPF treatment of BE(2)-M17 cells also led to the appearance of HMW COX IV immunobands (Fig. 5a red), as well as to the formation and accumulation of GM130 and STAT3 HMW complexes (Fig. 5b). When assessing VPF efficacy in targeting BE(2)-M17 cells, we detected < 10% viable BE(2)-M17 cells after 2 days of 5 µM VPF treatment, suggesting a potent and significant VPF-mediated BE(2)-M17 cell killing effect (Fig. 5c). We further tested VPF potency in another *MYCN-A* and predominantly YAP-negative NB cell line—the IMR-32 cell line. Similarly, we detected < 10% viable IMR-32 cells after 2 days of 5 µM VPF treatment, which underlines VPF potency and capacity to efficiently induce cell death in *MYCN-*A NB models shown to be predominantly YAP-negative (Supp. Figure 6). Altogether, these experiments suggest that VPF is a potent NB targeting agent, in both *MYCN-*A and *MYCN-*NA NB cells, independent of YAP/TAZ expression. At molecular level, we show that VPF-induced cell death across NB subtypes is commonly mediated via the early formation and accumulation of HMW complexes, which causes a severe impairment in cell homeostasis, oncogenic signaling and ultimately activation of cell stress and cell death mechanisms.

### VPF reduces NB survival and growth in vitro and in vivo

**Figure 6:**
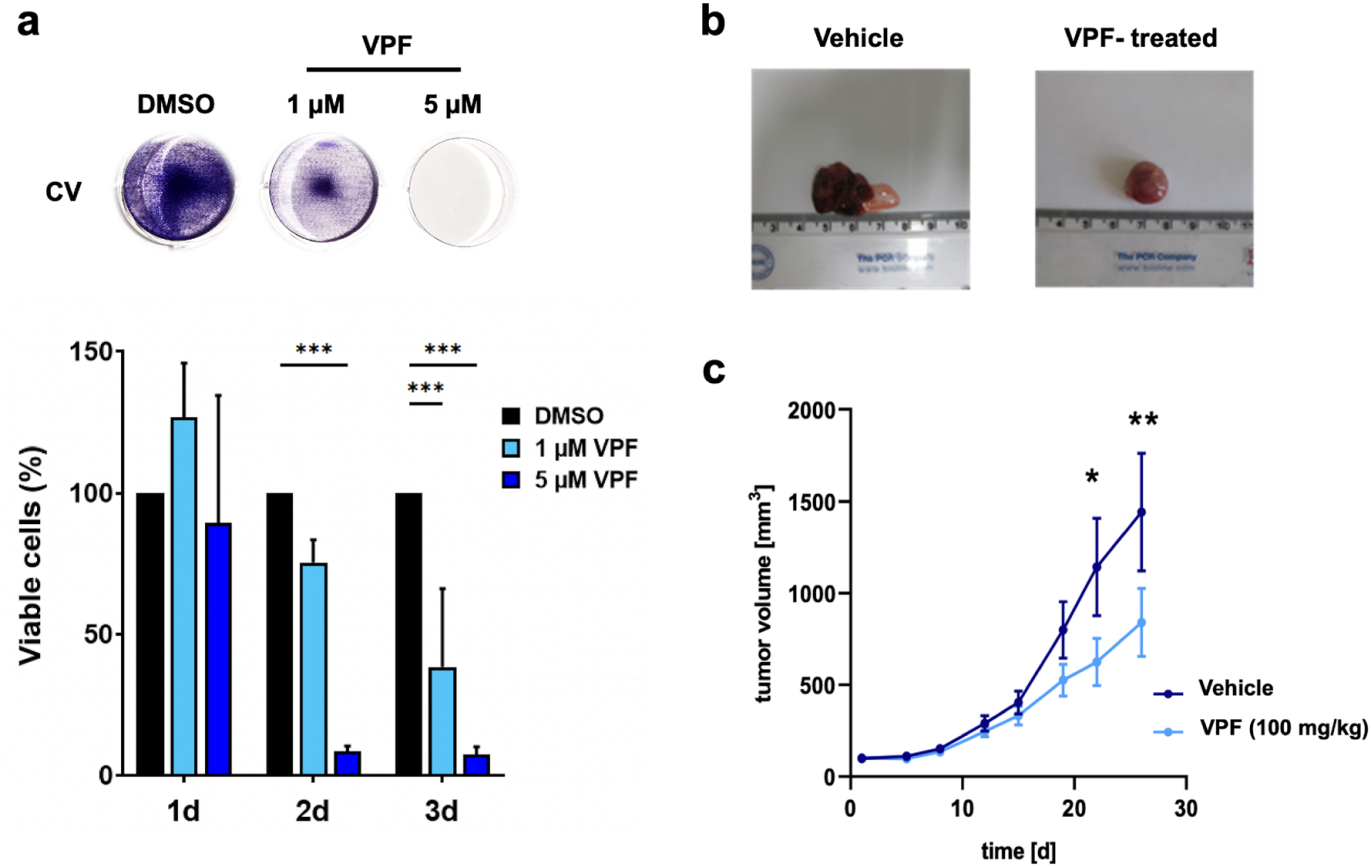
Verteporfin efficiently suppresses in vitro and in vivo SH-SY5Y neuroblastoma cell survival and tumor growth. (**a**) Crystal violet staining of viable SH-SY5Y cells after 1 to 3 days of 1 or 5 µM VPF treatment and the corresponding quantification graphs. Data is represented as average ± SEM (n = 4; normalized against DMSO controls set to 100%). Two-way analysis of variance (ANOVA) followed by Bonferroni *post-hoc* tests was used to assess the statistical significance (***p ≤ 0.001). (**b**) Representative images of SH-SY5Y NB xenografts isolated from mice treated with vehicle or 100 mg/kg VPF. (**c**) Graphical representation of tumor volume in control, vehicle-treated, group (dark blue) and 100 mg/kg VPF-treated group (light blue). Data represented as average ± SEM (n = 7 control group, n = 8 VPF-treated). Statistical significance was calculated using the two-way repeated measures ANOVA test (*p ≤ 0.05; **p ≤ 0.01).

We next investigated whether VPF-mediated impairment of neuroblastoma cell proliferation and survival in vitro translates into a tumor suppressive effect in vivo. For this purpose, we used the SH-SY5Y NB cell model, a well-established NB in vivo xenograft model^47^ and heterogenous neuroblastoma cell line containing distinct YAP- and N-MYC-expressing subpopulations (also see Fig. 1)^24^. Initially, we tested VPF potency to inhibit SH-SY5Y cell viability in vitro*.* We showed that VPF potently impairs SH-SY5Y cell viability in vitro, as indicated by < 10% viable cells detected 2 days following 5 µM VPF treatment (Fig. 6a). Next, to determine the potency of VPF in targeting NB in vivo, SH-SY5Y cells were injected subcutaneously into the right flanks of CD-1 nude mice. Once the xenografts reached 150 mm^3^ in size, VPF (100 mg/kg) or vehicle control (PBS) was injected intraperitoneally every second day. VPF treatment led to a significant decrease in tumor growth (Fig. 6b,c; Supp Fig. 7a), as well as a noticeable decrease in tumor weight (Supp Fig. 7b) compared to vehicle-treated mice. Moreover, VPF treatment was well tolerated in vivo, in line with previous reports^37,38,42^*,* with a slight reduction in murine body weight (Supp. Figure 7c). The individual tumor volume and body weight measurements are reported in Supp. Figure 7d–g. Altogether, the in vitro and in vivo SH-SY5Y results support that VPF exerts a suppressive effect on tumor growth in NB.

**Figure 7:**
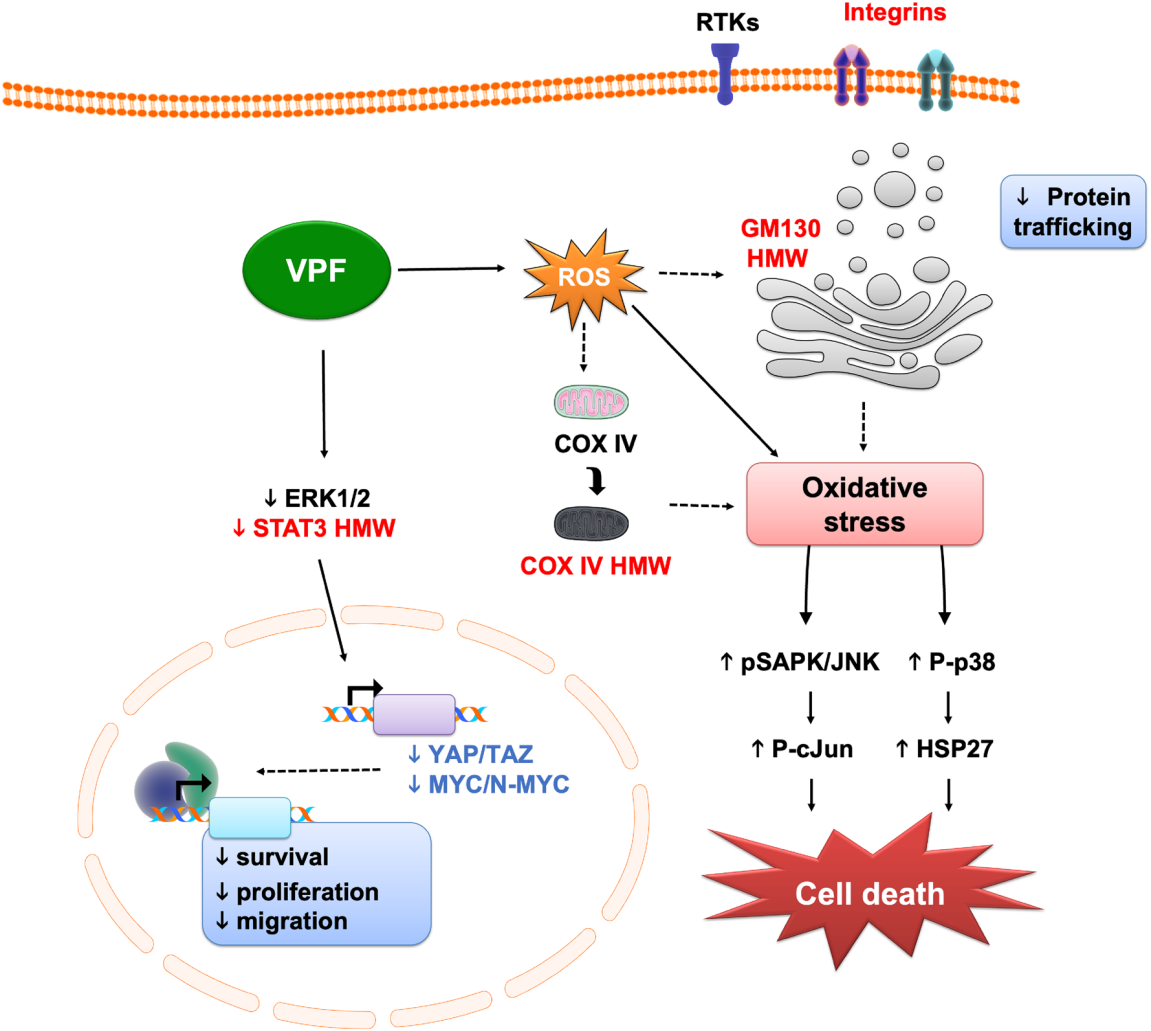
Verteporfin YAP-independent oncosuppressive effects in neuroblastoma. Schematic illustration of VPF-induced molecular and phenotypic effects, including downregulation of oncogenic factors—YAP/TAZ/MYC/N-MYC, oncogenic signaling cascades—ERK1/2/STAT3 signaling, and formation of high molecular weight complexes—HMW, leading to impaired cell proliferation, migration and survival, loss of cell homeostasis, activation of cell stress and cell death mechanisms. RTKs—Receptor Tyrosine Kinases; VPF—Verteporfin; ROS—Reactive Oxygen Species.

The VPF-induced NB antitumor effects identified in our study, including downregulation of oncogenic factors (YAP, TAZ, C-MYC, N-MYC, STAT3), formation of COX IV, GM130, STAT3 HMW complexes, disruption of cellular homeostasis, activation of cell stress and cell death effectors are presented in a summative schematic (Fig. 7).

## Discussion

Verteporfin (Visudyne®) is an FDA-approved photosensitizer clinically used in photodynamic therapy for age-related macular degeneration by targeting abnormal blood vessels^29^. Tumor growth, metastatic progression and therapy resistance are in part facilitated by tumor-associated neovasculature^48^. Therefore, the use of VPF as a targeting agent of tumor vasculature has been of high interest. Several pre-clinical and clinical studies reported VPF efficacy and safety in damaging tumor neovasculature, leading to tumor necrosis and inhibition of tumor growth, which ultimately increased the interest in investigating VPF potency as an anti-cancer therapeutic agent^42,49^. This is in line with our present study, where we also observed an anti-proliferative effect of VPF on neuroblastoma xenografts (Fig. 6b,c).

The anti-tumor effects of VPF are not limited to photoactivation. Liu-Chittenden et al. have shown that non-photoactivated VPF directly binds to YAP, preventing the formation of YAP-TEAD complexes, which limits YAP’s transcriptional activity^28^. The Hippo downstream effectors YAP/TAZ are commonly dysregulated in cancer and promote a wide range of oncogenic processes that regulate tumor growth, metastatic progression, and therapy-resistance^23,50^. Naturally, VPF efficiency in targeting YAP activity across different tumor entities has been investigated. Several studies showed that VPF-mediated YAP inhibition leads to a decrease in tumor cell proliferation, migration, and survival in vitro and suppression of tumor growth and progression in vivo^27,38,46,51^. With recent evidence of YAP/TAZ role in NB tumorigenicity, metastatic progression, treatment resistance and tumor relapse^13,14,17,18,52^, we aimed to investigate whether VPF can also be used as a potential therapeutic agent against NB.

We showed that YAP/TAZ-expressing NB cell models have a predominantly mesenchymal morphology and that YAP/TAZ expression generally overlaps with early NCC markers, but is absent in neuroblast or noradrenergic NB cells expressing DCX (Fig. 1). Of note, in early NCC cultures we observe a transient YAP-DCX overlap following the neural crest cell fate transition from neuroepithelial cells. Overall, YAP/TAZ expression positively correlates with C-MYC, but not N-MYC in both NB patient samples^4^ and across the cell lines tested (see Fig. 1a; Supp. Figure 1). We previously showed that YAP plays an important role during early neural crest stem cell development and that YAP^+^ NB cells do not respond to RA-induced differentiation^24,31^. Since VPF is a potent inhibitor of YAP activity^28^, we wanted to determine whether VPF can efficiently target NB subtypes that express YAP/TAZ. We used the metastatic GI-ME-N cell line as a representative NB model with high expression of YAP/TAZ. We showed that VPF is highly efficient in impairing cell viability (Fig. 2a,b,c,e) in GI-ME-N cells, as well as SK-N-AS cells—another YAP/TAZ positive NB cell line (Supp. Figure 2). At molecular level, we showed that VPF treatment of GI-ME-N cells resulted in downregulation of YAP/TAZ as well as C-MYC, which is in line with prior studies showing VPF-mediated YAP/TAZ downregulation and inhibition of transcriptional activity leading to impaired cell survival^15,27,46^. We also showed that mediators of cell proliferation, including CDK2,4,6 and cyclins, and pro-migratory factors, such as β1-integrin, FAK, CDC42, RAC1-3, were downregulated upon VPF treatment (Figs. 3, 4; Supp. Figure 4 a,b), also previously connected to VPF suppression of cell proliferation, migration, and tumor invasion^15,38,45,51^. Our observations in YAP-expressing NB lines align with the recent report by Fusco and colleagues, wherein in vitro VPF treatment of YAP/TAZ-expressing tumor initiating cells (TICs) derived from high-risk NB patients resulted in impairment in cell migration and induction of apoptosis^15^. However, it still remained unclear the mechanisms mediating VPF suppressive effects in NB and whether YAP expression was a necessity.

Interestingly, D551 fetal fibroblast cells, which highly express nuclear YAP, were able to tolerate VPF treatment and showed significantly less cytotoxicity compared to NB cells (see Fig. 2d,f). This observation upholds previous in vitro and in vivo studies suggesting a tumor-selective effect of VPF, wherein primary human hepatocytes, fibroblasts, normal intestine homeostasis, kidney, liver, and colon tissue were reported to tolerate VPF exposure^37,38,43^.

VPF light-independent tumor oncosuppressive mechanisms are not dependent nor limited to YAP/TAZ-expressing tumors^37^. In colorectal cancer (CRC) models, Zhang and colleagues have knocked-down YAP/TAZ expression and provided clear evidence that even in the absence of the Hippo effectors, VPF efficiently suppresses in vitro and in vivo CRC tumor growth^37^. Therefore, we also assessed in our NB models whether VPF suppression of NB cells is dependent on YAP/TAZ expression. We used YAP/TAZ GI-ME-N KO lines, as well as the BE(2)-M17 and IMR-32 cell lines—noradrenergic and predominantly YAP negative NB models, and demonstrated that even in the absence of YAP/TAZ expression VPF continues to be a potent NB targeting agent, causing significant cell-death effects (see Fig. 5; Supp. Figure 5; Supp. Figure 6). Moreover, to our knowledge, we provide first evidence of VPF impacting N-MYC expression, increasing its therapeutic potential and appeal not only in targeting *MYCN-A* and/or N-MYC expressing NB models as well as for other N-MYC-driven malignancies.

VPF antitumor effects can also be mediated via the formation of selective proteotoxic oligomeric complexes and direct inhibition of multiple kinases^29,35,37^. AlAmri and colleagues used a kinase assay and showed that VPF directly binds and inhibits the kinase activity of STE20/SPS1-related proline/alanine-rich kinase (SPAK) and oxidative-stress-responsive kinase1 (OSR1) involved in ion homeostasis. Moreover, eight additional kinases were also potently inhibited by VPF, including the inhibitor of nuclear factork-B kinase subunit epsilon (IKKe), mammalian STE20-like protein kinase 2 (MST2), germinal center kinase (GCK), mitogen-activated protein kinase kinase kinase kinase 3 (MAP4K3), mitogen-activated protein kinase 1 (MLK1), Unc-51-like autophagy activating kinase1 (ULK1), ULK2 and the lymphocyte-specific protein tyrosine kinase (Lck)^35^. We also observed kinase-activity related changes in our NB models following VPF treatment (see Supp. Figure 3; Figs. 3c–e, 4a, 5a), including decreased phosphorylation of cell homeostasis modulators (WNK1, HSP60), transcription (STAT members), protein synthesis (p70 S6 kinase), cell survival (AKT, ERK) and cell cycle (p27), and increased phosphorylation of cell stress transducers (p38, HSP27), adding to previously reported factors^35,37,38^. Zhang and colleagues have described the formation of HMW oligomerized protein complexes, particularly impacting p62 (a protein involved in autophagy) and the transcription factor STAT3, as a VPF tumor-selective proteotoxic mechanism^37^. VPF-induced proteotoxicity via HMW complexes was also confirmed in hepatocarcinoma, prostate cancer and other tumor cell lines^38,53^. Interestingly, while non-malignant cells can also uptake VPF, they are less sensitive to VPF proteotoxicity in part due to their ability to efficiently fight ROS generation and clear oligomeric protein complexes^37^. In contrast, malignant cells are less efficient in doing so, causing an accumulation of distinct HMW oligomeric complexes that become extremely cytotoxic, ultimately triggering a potent cell stress and cell death response^37,44^. We demonstrated that the formation of HMW complexes in NB cells is an early VPF-mediated tumor suppressive mechanism, as they appear as early as 30 min post-treatment and accumulate over time in both *MYCN*-NA and *MYCN*-A NB subtypes (see Figs. 3d, 4, 5a,b). While STAT3, p62 oligomeric complexes have been previously identified^37,38^, we additionally report the formation of COX IV (see Figs. 3d, 4a,c, 5a) and GM130 HMW oligomers in this context (Figs. 4b,c, 5b). COX IV is a subunit of the cytochrome-c oxidase involved in electron transfer and oxidative phosphorylation^54^, while GM130 is an essential Golgi protein involved in protein glycosylation and transport^55,56^. Interestingly, downregulation of GM130 expression and disruption of the Golgi apparatus were reported in endometrial cancer cells following VPF treatment^53^. These observations suggest that the formation of HMW oligomeric complexes is an early and central molecular mechanism of VPF-mediated tumor cell suppression, both in neuroblastoma and other tumor entities.

In addition to the in vitro evidence of VPF potency in targeting NB subtypes, we also provide evidence of its in vivo tumor-suppressive potential. For this purpose, we used the SH-SY5Y cell line, a heterogenous NB model with known tumorigenicity comprising of YAP^+^ and YAP^−^ subpopulations^24^, and frequently employed in NB differentiation and pre-clinical studies^31^. Indeed, using similar treatment conditions^38,46^, we showed that VPF impairs SH-SY5Y viability in vitro and reduces NB tumor growth in vivo (Fig. 6a–c; Supp. Figure 7).

Overall, this study presents solid evidence of VPF potency in targeting both *MYCN*-A and *MYCN*-NA NB models in a light and YAP-independent manner. However, further pre-clinical studies are needed to expand our knowledge on the best mode of administration, dosage, and potential combinatorial strategies to maximize VPF potency and limit treatment-related toxicities in NB patients. Possible scenarios of repurposing VPF as an anticancer agent are thus emphasized by its potent tumor-selective effects as well as its therapeutic versatility considering its administration (local or systemic), action (light-dependent or light-independent), molecular mechanisms mediating its oncosuppressive effects (YAP-dependent or independent, oxidative stress, proteotoxicity), as well as its sensitizing and synergistic potential^15,26,27,37–42,46,51,57,58^.

### Study limitations and future directions

The current study showed that VPF efficiently impairs NB cell viability via the formation of HMW proteotoxic complexes in both *MYCN-NA*/predominantly YAP-positive NB lines, as well as in *MYCN-A*/predominantly YAP-negative NB models, independent of YAP/TAZ expression and under non-light activating conditions. However, we are cognizant of several limitations, which we hope will be taken into consideration in follow-up studies. First, while we went to great efforts to minimize as best as we could light exposure of VPF-treated cells and samples, we cannot completely exclude a potential contribution by ambient light-mediated activation. Secondly, it would actually be exciting to also investigate the light-dependent therapeutic effects of VPF as a photodynamic agent in NB tumors. Thirdly, the select NB cell lines tested here were limited in number, therefore, additional in vitro and in vivo studies that will include a greater diversity of neuroblastoma cell models and, most importantly, patient samples would be impactful for a potential translation of VPF towards NB therapies. Finally, from a molecular perspective, the use of single cell multi-omics approaches to profile in vitro and in vivo VPF-treated patient samples may provide a most insightful strategy for unbiased identification and characterization of other potential targets and molecular mechanisms contributing to VPF’s oncosuppressive effects.

## Conclusion

High-risk NB tumors generally display an exceedingly aggressive and often therapy-resistant course. The presented cell and molecular analyses provide strong evidence that VPF, a clinically approved therapeutic agent, can efficiently induce cell death in both *MYCN-*A and *MYCN-*NA NB subtypes, independently of YAP/TAZ expression. Our data highlights the formation of high molecular weight oligomeric complexes as an early, shared, and central molecular mechanism of VPF-induced cell death across distinct NB cell models. In addition, we also provide the first in vivo indication of VPF’s potency in suppressing NB tumor growth. Altogether, our study underlines the therapeutic repurposing potential of VPF in NB and encourages further investigations on the use of VPF, or molecularly related analogues, as a therapeutic or adjuvant agent in NB treatment.

## Materials and Methods

### Cell lines and culture conditions

The human neuroblastoma SH-SY5Y and GI-ME-N cell lines were acquired from Leibniz Institute DSMZ-German Collection of Microorganisms and Cell Cultures, SK-N-AS and IMR-32 cell lines were obtained from the European Collection of Authenticated Cell Cultures (ECACC 94092302, 86041809, respectively) and the BE(2)-M17 cell line was acquired from the American Type Culture Collection (ATCC—Manassas, Virginia, United States). The SH-EP cell line was kindly provided by J. Roessler, Center for Pediatrics, Medical Center—University of Freiburg. Additional characteristics of the neuroblastoma cell lines used in this study are mentioned in Suppl. Table 1.

SH-SY5Y and BE(2)-M17 cells were maintained in 1:1 DMEM/F12 media (Gibco® Life Technologies) supplemented with 10% heat-inactivated fetal bovine serum (FBS; Gibco® Life Technologies), while GI-ME-N, SK-N-AS, SH-EP and IMR-32 cells were cultured in RPMI1640 media (Gibco® Life Technologies) supplemented with 10% FBS. Human embryonic stem cell-derived neural stem cells (hESC-NSCs) and neural crest cells (NCCs) were generated and maintained as previously described^24,59,60^. No antibiotics were added to the culture media. All cell cultures were maintained at 37 °C in a 5% CO_2_ humidified incubator.

### Short-term treatments

Cells were treated daily with verteporfin (VPF; Sigma) as indicated in the corresponding figure legends. Treatment solutions were prepared in normal growth media, if not indicated otherwise. Given the high photosensitivity and light-activation effects of VPF, both direct and ambient light exposure of VPF-treated cells was minimized as much as possible: VPF treatments were performed in the dark (no lights in the tissue culture hood, if possible, the room was darkened as well), no microscopy-light exposure of treated cells, the culture plates and cell pellets were covered and protected from direct light exposure.

### Western blot analysis

Cells were lysed in either sample buffer (0.4 M Tris–HCl pH 6.8; 6% SDS; 30% glycerol) or in NP-40 lysis buffer (20 mM Tris–HCl pH8, 137 mM NaCl, 10% glycerol, 1% NP-40, 2 mM EDTA, 1 mM freshly-added phenylmethylsulphonyl fluoride—PMSF). Protein immunoblotting was performed as previously described^24^. Briefly, 10–15 µg/ml of total protein lysate were subjected to SDS–polyacrylamide gel electrophoresis (Mini-PROTEAN ® TGX™ precast gels; BioRad), then transferred onto PVDF membrane (Merck-Millipore). The membranes were blocked in a 5% BSA-TBST solution (TBS with 0.1% Triton-X), for 1 h at room-temperature (RT) or overnight (O/N) at 4 °C, on a rocking platform. After blocking and prior to primary antibody hybridization, the membranes were cut to enable multi-protein probing in samples with limited protein concentrations. Primary antibody incubation was done for at least 1 h at RT or O/N at 4 °C with specific primary antibody solutions. Unbound antibodies were removed by repeated washing steps in 1% TBST solution. Subsequently, the membranes were incubated in the corresponding HRP-conjugated secondary antibody solutions. Immunodetection of target proteins was done using the WesternBright Sirius kit (Advansta) and an ImageQuant LAS 400 mini reader (GE Healthcare). Antibodies and used concentrations are indicated in Suppl. Table 2. Uncropped western blot images are included in Suppl. Figure 8.

### Indirect immunofluorescence analysis

Cells were fixed for 20 up to 30 min at RT in 4% PFA solution, then repeatedly washed before permeabilization in a 0.5% Triton-X solution. The cells were incubated for 1 h in blocking solution, then stained O/N at 4 °C with specific primary antibodies. Unbound primary antibodies were removed by PBS washing before proceeding with secondary antibody staining and nuclei counterstaining (Hoechst 33342 1:10,000; Life Technologies). After removing the unbound secondary antibodies, the samples were mounted in ProLong Diamond Antifade Mountant (Life Technologies). Images were acquired using a ZEISS AxioImagerM2 fluorescence microscope and post-processed using the Zen Blue software version 1.1.2.0 (Zeiss).

### Transwell migration assay

We seeded 5 × 10^4^ GI-ME-N cells per insert (8 µm, ThinCert™ Greiner Bio-One) in 200 µl 1% FBS-supplemented media containing DMSO or VPF. In the lower chamber, 800–1000 µl of 10% FBS media were used as a chemoattractant. The cells were allowed to migrate for 22 h, after which a cotton swab was used to remove the non-migrating cells from the upper chamber. The inserts were then washed in PBS before fixation for 20 min, at RT in 4% PFA solution. Following repeated washing steps for PFA removal, the migrating cells were stained for 30 min with 0.1% Crystal violet solution (Chroma-Gesellschaft Schmid & Co.). The inserts were washed and left to air-dry before being imaged using a ZEISS AxioImagerM2 fluorescence microscope. For the quantitative measurement of migrating cells, the crystal violet dye was extracted by incubating the membranes in a 10% acetic acid solution for 20 min, on a rocking platform at RT. The extracted dye was then spectrophotometrically measured at 595 nm. The transwell migration assay was performed in technical duplicates and repeated at least three times.

### In vitro scratch assay

Cell monolayers were scratched using a sterile 200 µl pipette tip and 2–3 random visual fields per treatment condition were imaged at the indicated time points. Phase contrast images were acquired using an Axiovert 40 CFL inverted microscope and processed using the ImageJ software version 1.50e.

### Cell viability assay

1.25 × 10^4^ GI-ME-N, 2.5 × 10^4^ SK-N-AS cells/cm^2^, BE(2)-M17 cells/cm^2^, 5 × 10^4^ SH-SY5Y cells/cm^2^ or 7.5 × 10^4^ IMR-32 cells/cm^2^, were seeded in 12 well plates (Corning™ Falcon™) and treated daily for 1 to 3 consecutive days, as indicated in the corresponding figure legends. Additionally, the GI-ME-N KO lines were seeded at 3.2 × 10^4^ cells/cm^2^ in 48 well plates (Thermo Fisher) and treated daily for three consecutive days with the indicated compounds. For crystal violet staining, cells were washed, fixed in 4% PFA and then stained with 0.1% Crystal violet solution for 30 min at RT to assess the viable cells. The stained cells were washed repeatedly to remove excess dye and left to air-dry at RT, before scanned images were documented. For quantitative assessment of cell viability, the retained crystal-violet dye was extracted, using a 10% acetic acid solution and 20 min incubation at RT, and measured at 595 nm using a VICTOR™ X Multilabel Plate Readers (PerkinElmer Inc.) or a TECAN Spark multiplate reader (Tecan Trading AG).

### CRISPR/Cas9-generated NB knock-out lines

For targeted knock-out (KO), cells were transfected with commercially available CRISPR/Cas9 double nickase plasmid constructs (Santa Cruz Biotechnology; see Suppl. Table 3) using the DharmaFECT™ Transfection Reagents (Dharmacon Inc.) in accordance with the manufacturer’s guidelines. The control line was generated using a double nickase plasmid encoding for the D10A mutated Cas9 nuclease and a non-targeting 20 nt scramble guide RNA (Santa Cruz Biotechnology; sc-437281). 48 h post-transfection, the cells were harvested and FACS-sorted to generated single-cell GFP-positive clones. After clonal expansion, the targeted KO efficacy was assessed by western blot and/or immunofluorescence.

### Mouse xenograft models

Animal experiments were approved by the Austrian ministry of education, Science and research (No. 2020-0.148.799). The mice were kept in the animal facility of the Paracelsus Medical University Salzburg, Austria under pathogen-free conditions. Water and food were supplied ad libitum*.* For the generation of SH-SY5Y xenografts, 2 × 10^7^ cells in 0.2 ml of a 50:50 medium/Matrigel (BD Matrigel Matrix 356234) mixture were subcutaneously injected in the right flanks of 5- to 6-week-old female CD-1 nude mice (Charles River, Sulzfeld Germany). SH-SY5Y is a heterogenous neuroblastoma cell line that includes both YAP + /N-MYC-, as well as YAP-/N-MYC + cells, with a well-established tumor formation potential in vivo^47^. Once tumors reached 150 mm^3^ in size, 100 µl of PBS (vehicle control) or 100 mg/kg VPF (eNovation Chemicals LLC D480571) solution in PBS was injected intraperitoneally every second day for maximum 26 days. Tumor volume and body weight were measured twice a week. Tumor size was determined using a Vernier caliper and calculated based on the formula for the tumor volume: W = (length * width * height) / 2. Mice were euthanized either when termination criteria were met (health status, tumor ulceration or tumor size > 10% body weight) or on day 28.

### Ethical approval

All animal experiments and procedures were conducted in accordance with the Salzburg Animal Care and Use Committee and the ARRIVE guidelines. The mice were maintained under specific pathogen-free conditions and care as required by the Austria Act on Animal Experimentation. Animal experiments were approved by the Austrian ministry of Education, Science and Research (No. 2020-0.148.799).

## Supplementary Information

Supplementary Information.

## Data Availability

All datasets analyzed and included in the current study are available from the corresponding author on reasonable request.
