# Supplementary material for: Verteporfin-induced proteotoxicity impairs cell homeostasis and survival in neuroblastoma subtypes independent of YAP/TAZ expression
Source: Sci Rep. 2023 Mar 7;13:3760. doi: 10.1038/s41598-023-29796-2 (PMC9992669; doi:10.1038/s41598-023-29796-2)

Supplementary materials for:

Verteporfin-induced proteotoxicity impairs cell homeostasis and survival in neuroblastoma subtypes independent of YAP/TAZ expression

Alexandra-Larisa Condurat[1,2], Sepideh Aminzadeh-Gohari[3], Mirjana Malnar[4,5], Nicole Schider[4,5], Leonie Opitz[4,5], Ria Thomas[1,2], Vishal Menon[1,2], Barbara Kofler[3], Jan Pruszak[1,4,5§]

[1] Emmy Noether-Group for Stem Cell Biology, Department of Molecular Embryology, Institute of Anatomy and Cell Biology, Faculty of Medicine, University of Freiburg, Freiburg, Germany
[2] Spemann Graduate School of Biology and Medicine and Faculty of Biology, University of Freiburg, Freiburg, Germany
[3] Research Program for Receptor Biochemistry and Tumor Metabolism, Department of Pediatrics, University Hospital of the Paracelsus Medical University, Salzburg, Austria
[4] Institute of Anatomy and Cell Biology, Salzburg, Paracelsus Medical University, Salzburg, Austria
[5] Center of Anatomy and Cell Biology, Salzburg and Nuremberg, Paracelsus Medical University, Salzburg, Austria
[§]correspondence: jan.pruszak@pmu.ac.at

Supplementary Materials and Methods

Human Protein-kinase profile

The Proteome Profiler Human Phospho-Kinase Array Kit (ARY003B; R&D Systems Inc.) was used to investigate the protein kinase phosphorylation profiling of GI-ME-N cells treated for 24 h with 5 µM VPF. The cells were lysed and prepared for analysis according to the manufacturer's protocol. A total of 400 µg protein lysate were used per array set. The immunodetection of target proteins was done using the WesternBright Sirius kit (Advansta) and an ImageQuant LAS 400 mini reader (GE Healthcare). The signal intensity measurement for quantitative analysis was done using the ImageJ software version 1.50e.

Supplementary Table

Suppl. Table 1: Origin and characteristics of *MYCN*-non-amplified and *MYCN*-amplified metastatic neuroblastoma cell lines used in this study (*atcc.org*)

Cell line	Characteristics
**GI-ME-N**	• Origin: bone marrow metastasis of a 2 years old female<br>• 6 months chemotherapy (poor response, very aggressive)
**SK-N-AS**	• Origin: bone marrow metastasis biopsy – 6 years old female<br>• Histology: poorly differentiated embryonal neuroblastoma
**SH-EP**	• Subclone of SH-SY5Y cell line (enriched for the "S"-subtype cells)<br>• Origin: bone marrow metastasis biopsy – 4 years old female
**SH-SY5Y**	• thrice-cloned sub-line of SK-N-SH cell line<br>• Origin: bone marrow metastasis biopsy – 4 years old female<br>• Patient underwent repeated rounds of radiation and chemotherapy
**BE(2)-M17**	• a twice-sub-cloned cell line derived from the SK-N-BE(2) cell line<br>• Origin: bone marrow metastasis biopsy – 22 months old male<br>• Patient had repeated courses of chemotherapy and radiotherapy
**IMR-32**	• Origin: abdominal metastasis biopsy – 13 months old male<br>• Histology – neuroblastoma with rare areas of organoid differentiation

Suppl. Table 2: List of antibodies used in this study and the assay-specific applied concentrations

Antibody	Company	Catalogue #	WB	IF
**Atg16L1**	Cell Signaling Technology	8089	1000	**100**
**BECLIN-1**	Cell Signaling Technology	3495	1000	**-**
**CDC42**	Cell Signaling Technology	2466	1000	**-**
**CDK2**	Cell Signaling Technology	2546	1000	**-**
**CDK4**	Cell Signaling Technology	12790	1000	**-**
**CDK6**	Cell Signaling Technology	3136	2000	**-**
**c-Jun**	Cell Signaling Technology	9165	1000	**-**
**Cleaved Caspase-3**	Cell Signaling Technology	9664	1000	**400**
**Cleaved PARP**	Cell Signaling Technology	5625	1000	**400**
**C-MYC**	Cell Signaling Technology	13987	1000	**500**
**COX IV**	Cell Signaling Technology	4850	1000	**-**
**Cyclin D1**	Cell Signaling Technology	2978	1000	**-**
**Cyclin D3**	Cell Signaling Technology	2936	1000	**-**
**DCX**	Santa Cruz Biotechnology	sc-8066	500	**200**
**FAK**	Cell Signaling Technology	13009	1000	**-**
**FOXD3**	Cell Signaling Technology	2019	1000	**-**
**GAPDH**	Santa Cruz Biotechnology	sc-59540	5000	**-**
**GM130**	Cell Signaling Technology	12480	1000	**-**
**ILK1**	Cell Signaling Technology	3856	1000	**-**
**Integrin α4**	Cell Signaling Technology	8440	1000	**-**

Antibody	Company	Catalogue #	WB	IF
**Integrin β1**	Cell Signaling Technology	9699	1000	**-**
**Integrin β3**	Cell Signaling Technology	13166	1000	**-**
**Integrin β4**	Cell Signaling Technology	4707	1000	**-**
**Ki67p**	Leica	NCL-Ki67p	1000	**1000**
**LC3A/B**	Cell Signaling Technology	12741	1000	**100**
**N-Cadherin**	Cell Signaling Technology	13116	1000	**-**
**NCAM**	Santa Cruz Biotechnology	sc-106	200	**200**
**NF2**	Santa Cruz Biotechnology	sc-332	200	**-**
**N-MYC**	Santa Cruz Biotechnology	sc-53993	500	**400**
**N-MYC**	Cell Signaling Technology	51705	1000	**-**
**p-Akt (Ser473)**	Cell Signaling Technology	4060	500	**-**
**Pan-TEAD**	Cell Signaling Technology	13295	1000	**-**
**PAX3**	Cell Signaling Technology	12412	1000	**-**
**P-c-Jun**	Cell Signaling Technology	3270	1000	**500**
**PERIPHERIN**	Santa Cruz Biotechnology	sc-7604	200	**-**
**p-ERK 1/2 (p44/42)**	Cell Signaling Technology	4695	1000	**-**
**pFAK (Y397)**	Abcam	ab39967	600	**150**
**P-HSP27**	Cell Signaling Technology	9709	1000	**200**
**P-p38 MAPK**	Cell Signaling Technology	4511	500	**-**
**p-p44/42**	Cell Signaling Technology	4370	500	**200**
**P-SAPK/JNK**	Cell Signaling Technology	4668	1000	**-**
**Rac1/2/3**	Cell Signaling Technology	2465	1000	**-**
**SOX10**	Cell Signaling Technology	89356	1000	**-**
**SOX9**	Cell Signaling Technology	82630	1000	**-**
**ß-CATENIN**	Cell Signaling Technology	8480	1000	**100**
**STAT3**	Cell Signaling Technology	9139	1000	**-**
**TAZ**	Cell Signaling Technology	70148	1000	**-**
**TAZ**	Cell Signaling Technology	4883	700	**-**
**TH**	Pel-Freez Biologicals	40101	500	**-**
**TUJ1**	BioLegend	PRb-435P	5000	**1000**
**TUJ1**	Covance	MMs-435P	1000	**1000**
**VIMENTIN**	BD Pharmingen™	550513	1000	**500**
**YAP**	Cell Signaling Technology	14074	1000	**100**
**YAP/TAZ**	Santa Cruz Biotechnology	sc-101199	200	**100**
**CD44**	eBiosciences	17-0441-82	-	**1:50**
**CD29**	eBiosciences	11-0299-42	-	**1:50**

Suppl. Table 3: Guide RNA sequences for CRISPR/Cas9 -mediated knock-out

Target	Santa Cruz Biotechnology #	gRNAs sequence
YAP	sc-400040-NIC	gRNA1: GGCGACCCAGGCGGCGCCGC<br>gRNA2: GCCGGTTGCCCGGGTCCGGA
TAZ	sc-400320-NIC	gRNA1: TCCAGCACCGACTCGTCGGG<br>gRNA2: CGCGAGTGCGAGCCCGAATC

Supplementary Figures

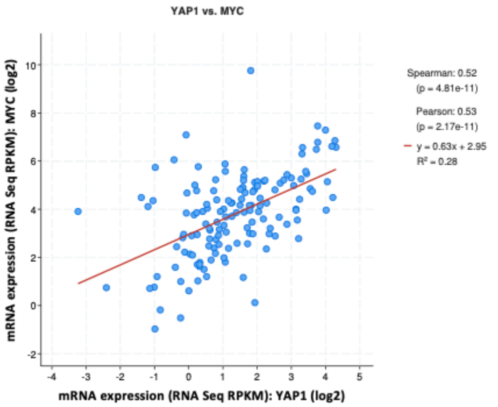

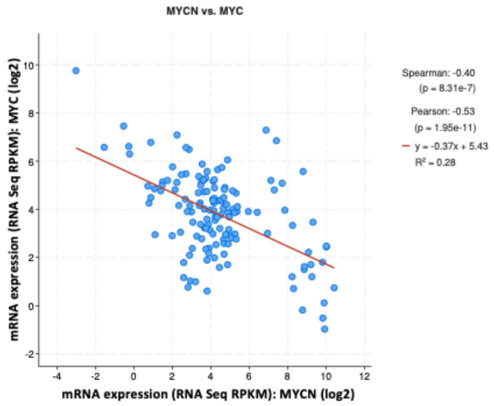

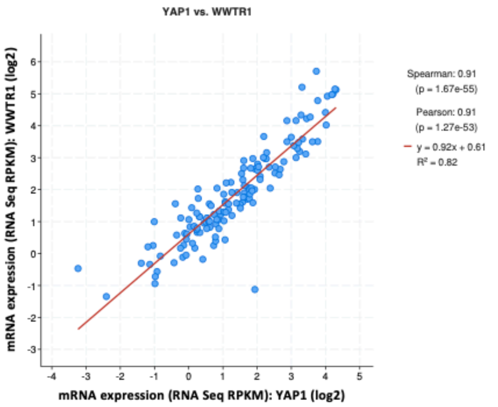

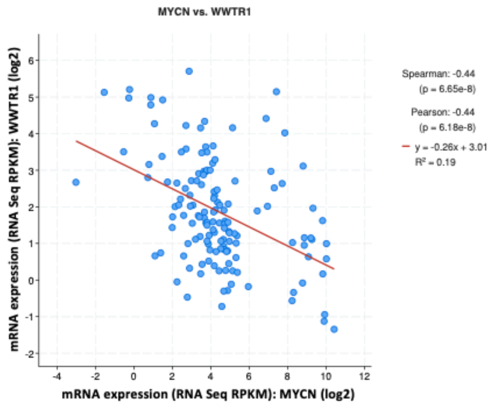

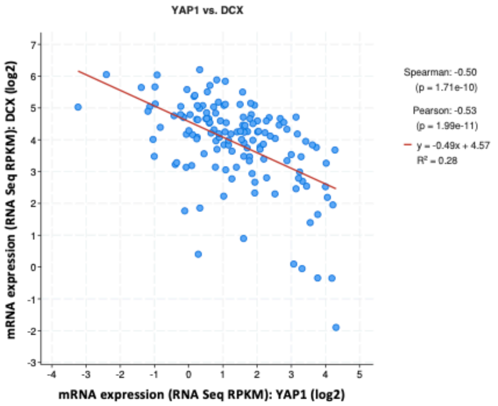

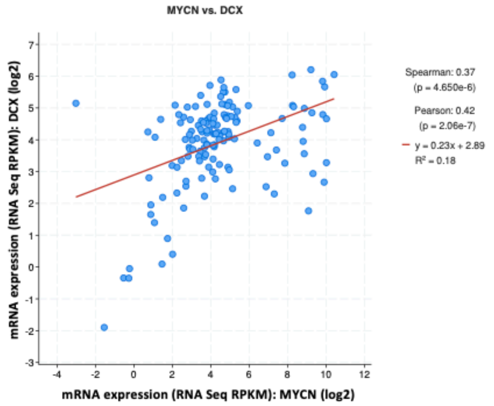

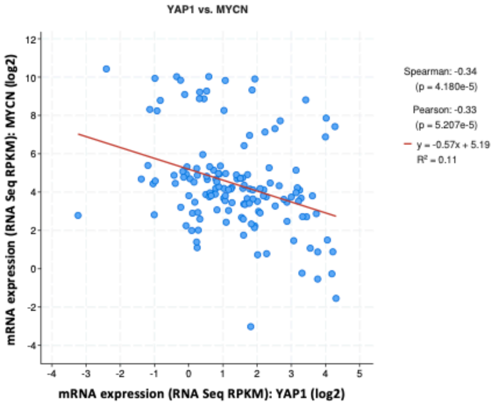

Supplementary Figure 1: *YAP1* expression positively correlates with *MYC* expression and negatively correlates with *MYCN* expression. Scatter plots illustrating the correlation between *YAP1-MYC-MYCN-WWTR1-DCX* gene expression in 143 bulk RNA sequenced NB patient samples. The results presented are in whole based upon data generated by the Therapeutically Applicable Research to Generate Effective Treatments (https://ocg.cancer.gov/programs/target) initiative, phs000467, available at https://portal.gdc.cancer.gov/projects. The figures were generated using the cbioportal.org platform.

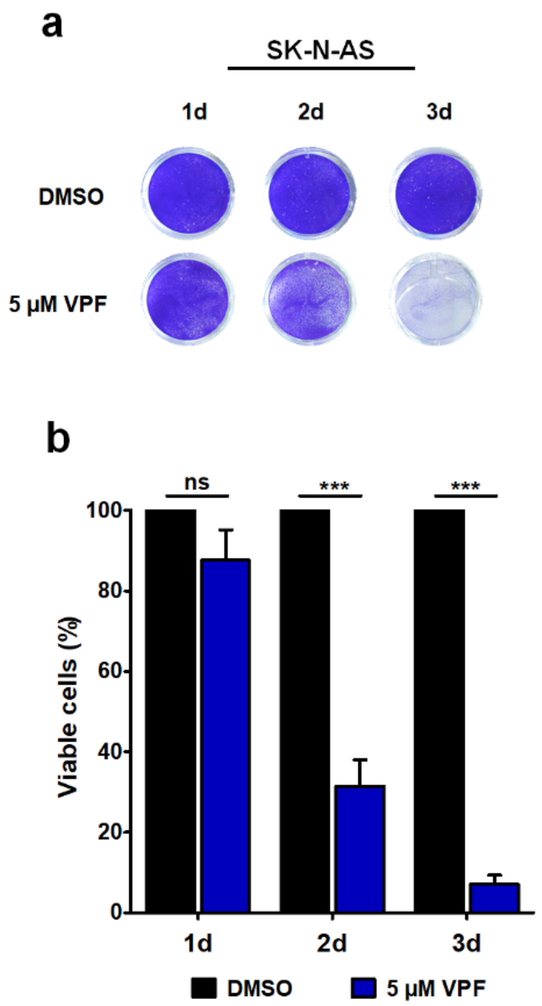

Supplementary Figure 2: Verteporfin efficiently targets YAP-positive SK-N-AS NB cells. (**a**) Representative images of crystal violet staining of viable SK-N-AS cells following 1 to 3 days of 5 µM verteporfin (VPF) treatment and (**b**) the corresponding quantification of viable cells. Data is represented as average ± SEM (n = 4; normalized against DMSO controls set to 100%), Two-way analysis of variance (ANOVA) followed by Bonferroni *post-hoc* test was used to assess the statistical significance (ns = not significant; ***$p \leq 0.001$).

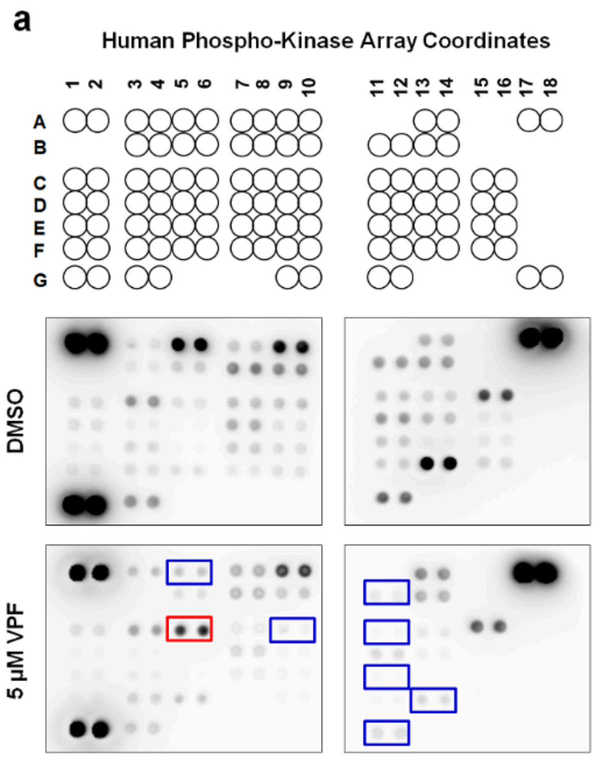

b

#	Target	Phosphorylation site	Fold change
A1-2	Reference	-	-1.03
A3-4	p38α	T180/Y182	2.50
A5-6*	ERK1/2	T202/Y204, T185/ Y187	-6.19
A7-8	JNK 1/2/3	T183/Y185, T221/ Y223	1.80
A9-10	GSK-3α/β	S21/S9	1.09
A13-14	p53	S392	1.87
A17-18	Reference	-	1.01
B3-4	EGF R	Y1086	-1.15
B5-6	MSK1/2	S376/S360	-1.31
B7-8	AMPKα1	T183	-1.03
B9-10	Akt 1/2/3	S473	-1.66
B11-12*	Akt 1/2/3	T308	-2.99
B13-14	p53	S46	-1.15
C1-2	TOR	S2448	1.28
C3-4	CREB	S133	1.04
C5-6#	HSP27	S78/S82	9.34
C7-8	AMPKα2	T172	-1.91
C9-10*	β-Catenin	-	-2.76
C11-12*	p70 S6 Kinase	T389	-3.03
C13-14	p53	S15	-1.61
C15-16	c-Jun	S63	1.14
D1-2	Src	Y419	-1.97
D3-4	Lyn	Y397	-1.19
D5-6	Lck	Y394	1.03
D7-8	STAT2	Y689	-1.69
D9-10	STAT5a	Y694	-1.80
D11-12	p70 S6 Kinase	T421/S424	-2.14
D13-14	RSK1/2/3	S380/S386/S377	-2.84
D15-16	eNOS	S1177	-2.66
E1-2	Fyn	Y420	1.23
E3-4	Yes	Y426	-1.57
E5-6	Fgr	Y412	-1.79
E7-8	STAT6	Y641	-1.83
E9-10	STAT5b	Y699	-1.45
E11-12*	STAT3	Y705	-3.01
E13-14	p27	T198	-8.15
E15-16	PLC-γ1	Y783	-4.33
F1-2	Hck	Y411	1.05
F3-4	Chk-2	T68	1.67
F5-6	FAK	Y397	2.21
F7-8	PDGF Rβ	Y751	-1.91
F9-10	STAT5a/b	Y694/Y699	-2.04
F11-12	STAT3	S727	-2.33
F13-14*	WNK1	T60	-8.40
F15-16	PYK2	Y402	-2.65
G3-4	PRAS40	T246	-1.45
G11-12*	HSP60	-	-3.33

Supplementary Figure 3: Verteporfin impacts the phosphorylation profile of multiple kinases, particularly those regulating cell homeostasis and cell stress. (**a**) Representative blots of a Human Phospho-Kinase Array of GI-ME-N cells treated for 24h with 5 µM VPF or DMSO (vehicle control) highlighting >2 fold-changes in the phosphorylation profile of HSP27, pERK1/2, p27, WNK1, PLC-γ1, HSP60, AKT, STAT3, p70 (n=2). (**b**) Table showing the fold change in the phosphorylation profile of indicated targets following 24h treatment of GI-ME-N cells with DMSO or 5 µM VPF. * = blue rectangle, # = red rectangle.

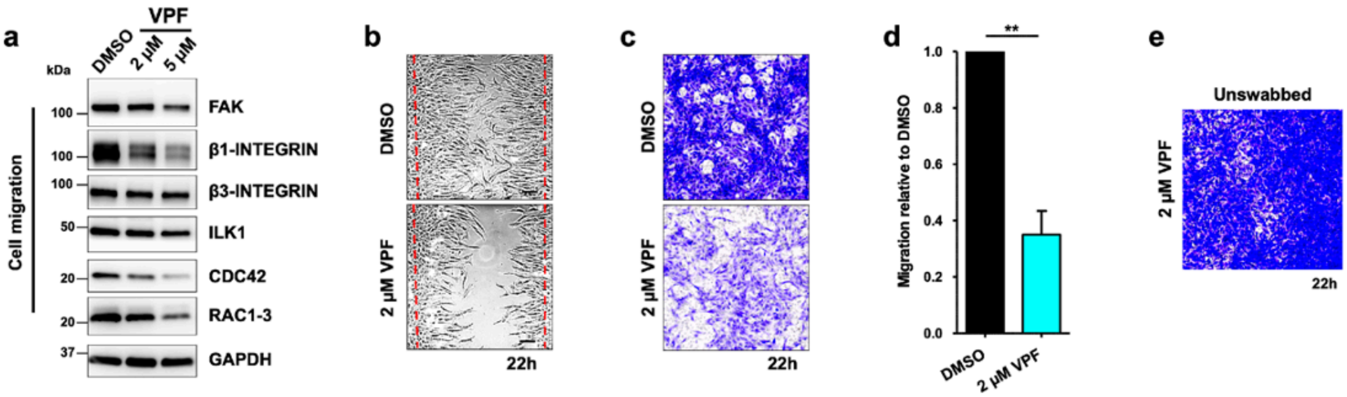

Supplementary Figure 4: Verteporfin impairs cell migration in GI-ME-N cells. (**a**) Western blot analysis of GI-ME-N cells treated for 24h with either 2 or 5 µM VPF shows downregulation of pro-migratory factors FAK, β1-integrin, CDC42, RAC1-3, compared to the DMSO control. Representative phase contrast images of an *in vitro* scratch assay (**b**) and a transwell migration assay (**c**) at 22h post 2 µM VPF treatment of GI-ME-N cells. Both migration assays show a reduction in the migratory potential of GI-ME-N cells upon treatment with VPF. (**d**) Quantification of migrating GI-ME-N cell treated with 2 µM VPF, assessed by transwell migration assay and quantified using crystal violet staining. Bars represent average ± SEM (n = 4; normalized against DMSO controls set to 1.0), statistical significance was determined using a one sample t-test: $^{**}p \leq 0.01$. Scale bar: 100 µm. **e**) Bright-field image of GI-ME-N cells remaining on the upper part of an unswabbed transwell membrane at 22h following 2 µM VPF treatment, confirming that the observed reduction in migration is not caused by impairment in cell survival.

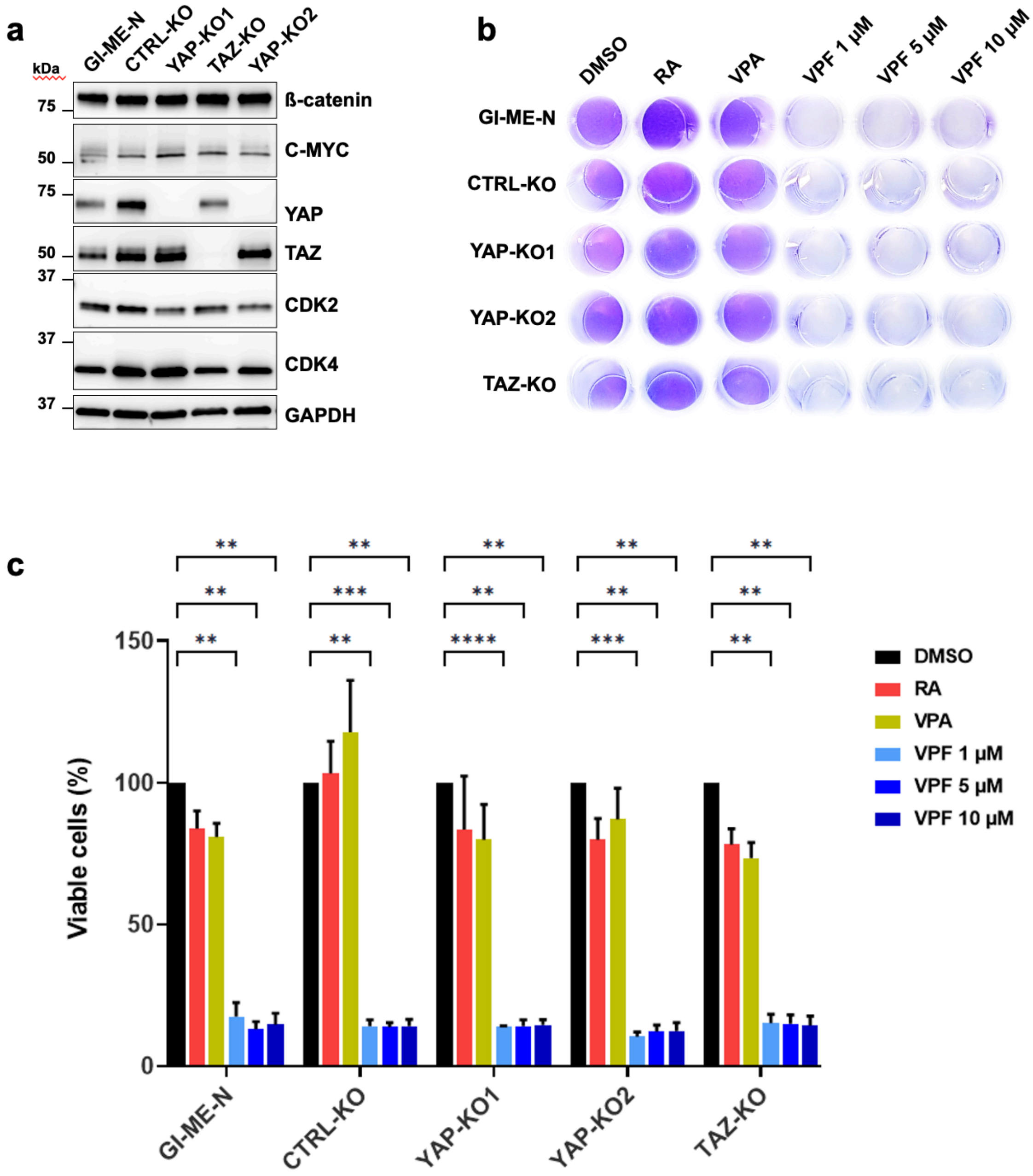

Supplementary Figure 5: YAP/TAZ expression is not required for verteporfin-induced cell death in neuroblastoma cells. (**a**) Immunoblot characterization of CRISPR/Cas9 nickase-generated YAP and TAZ knock-out (KO) GI-ME-N NB lines. Crystal violet staining images (**b**) and corresponding quantification (**c**) of parental GI-MEN, control (CTRL), YAP and TAZ KO lines treated daily for 3 consecutive days with vehicle control (DMSO), retinoic acid (RA; 10 µM), valproic acid (VPA; 2 mM) or verteporfin (VPF; 1 µM, 5 µM or 10 µM). Bars represent average ± SEM (n = 3; normalized against DMSO controls set to 100%). Statistical significance was determined using a two-way analysis of variance (ANOVA) with Dunnett's *post-hoc* test: **p ≤ 0.01; ***p ≤ 0.001; ****p ≤ 0.0001.

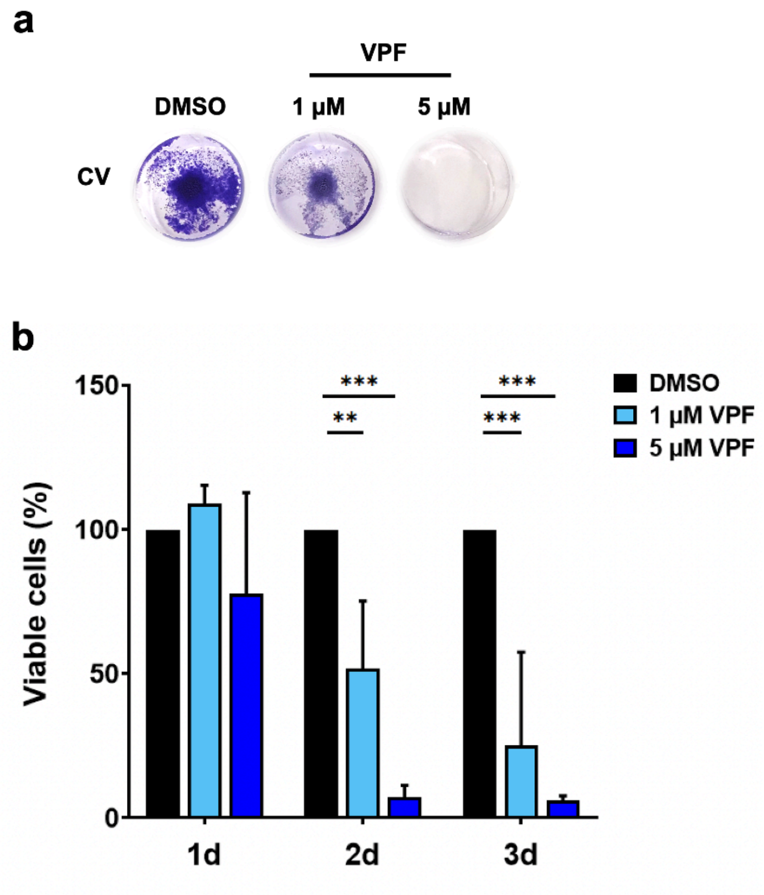

Supplementary Figure 6: Verteporfin efficiently targets YAP-negative IMR-32 neuroblastoma cells. (**a**) Representative images of crystal violet staining of viable IMR-32 cells following 1 to 3 days with 1 µM or 5 µM verteporfin (VPF) treatment and (**b**) the corresponding quantification of viable cells. Data is represented as average ± SEM (n = 4; normalized against DMSO controls set to 100%). Two-way analysis of variance (ANOVA) followed by Bonferroni *post-hoc* test was used to assess the statistical significance (**$p \leq 0.01$; ***$p \leq 0.001$).

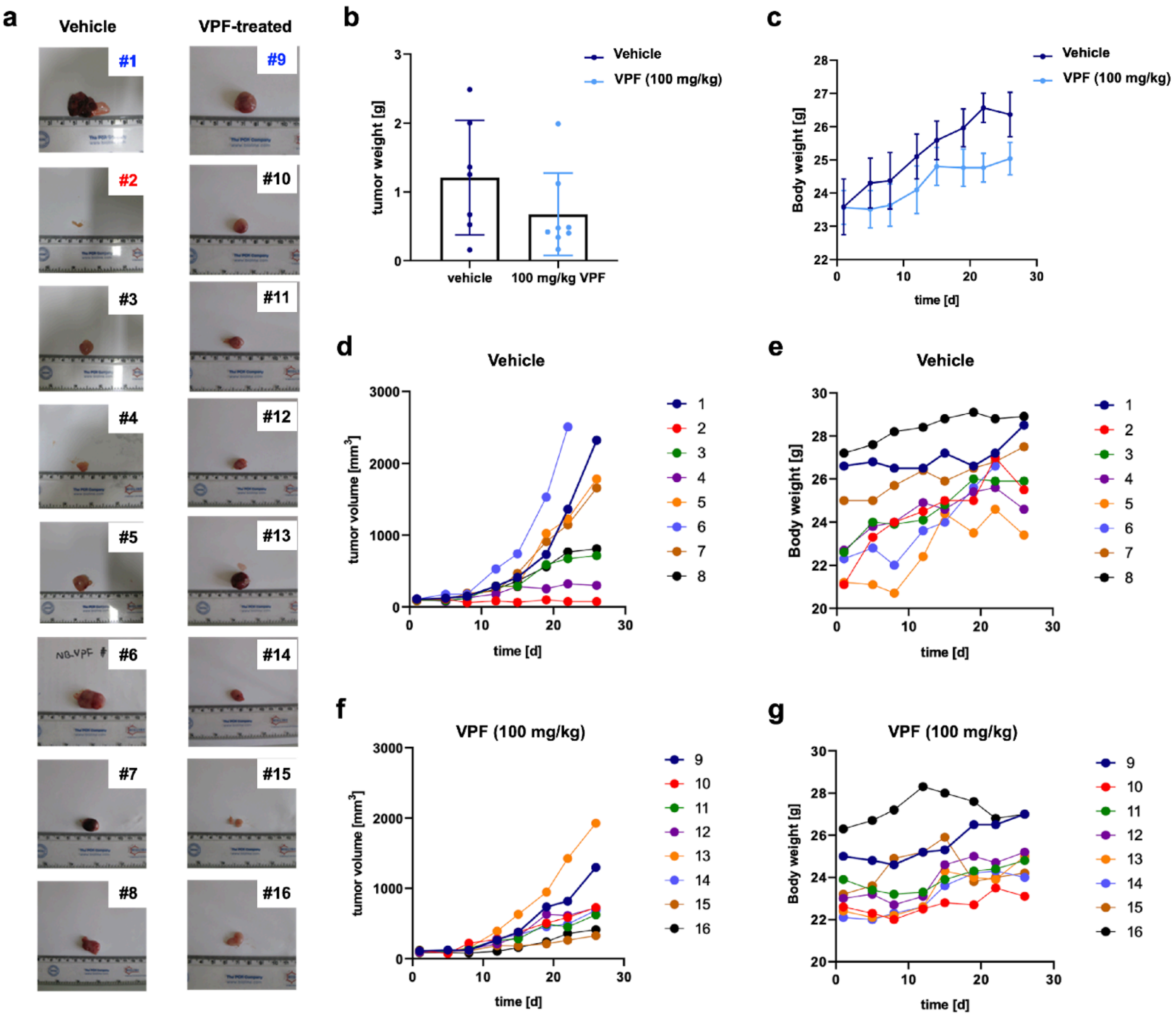

Supplementary Figure 7: Verteporfin reduces SH-SY5Y *in vivo* tumor growth. (**a**) Images of tumors isolated from vehicle control vs VPF-treated mice. In blue are marked images included in main Fig. 6, in red is marked xenograft #2 from the vehicle group that failed to engraft, therefore was excluded from the comparative statistical analysis. Graphical representation of tumor weight (**b**) and body weight (**c**) in control - vehicle treated group (**dark blue**) and 100 mg/kg VPF-treated group (**light blue**). Data represented as average ± SEM (n =7* control group, n = 8 VPF–treated). Individual measurements of tumor volume and bodyweight of vehicle-treated mice (**d, e**) or 100 mg/kg VPF-treated mice (**f, g**), respectively. Measurements were taken twice per week.

Supplementary Figure 8: Uncropped western blot images. Black boxes represent membrane borders as membranes were cut post-blocking and prior to specific primary antibody solution probing. Red boxes indicate the western blot area included in the main or supplementary figures. Grey rectangular shapes are placed over western blot images of protein immunoblots and/or samples not part of this study, but concurrently imaged.

Figure 1 western blot images

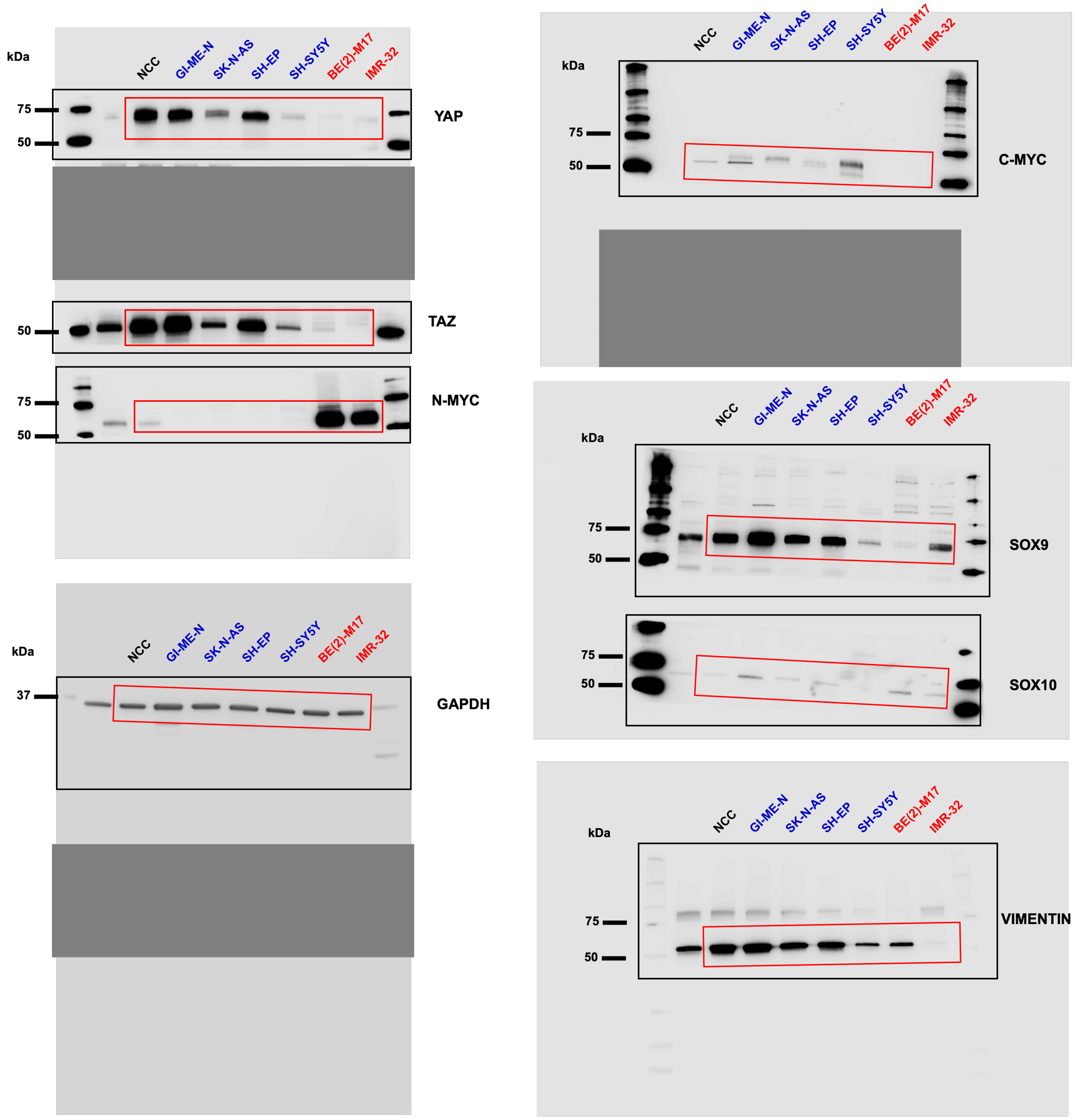

Figure 1 western blot images

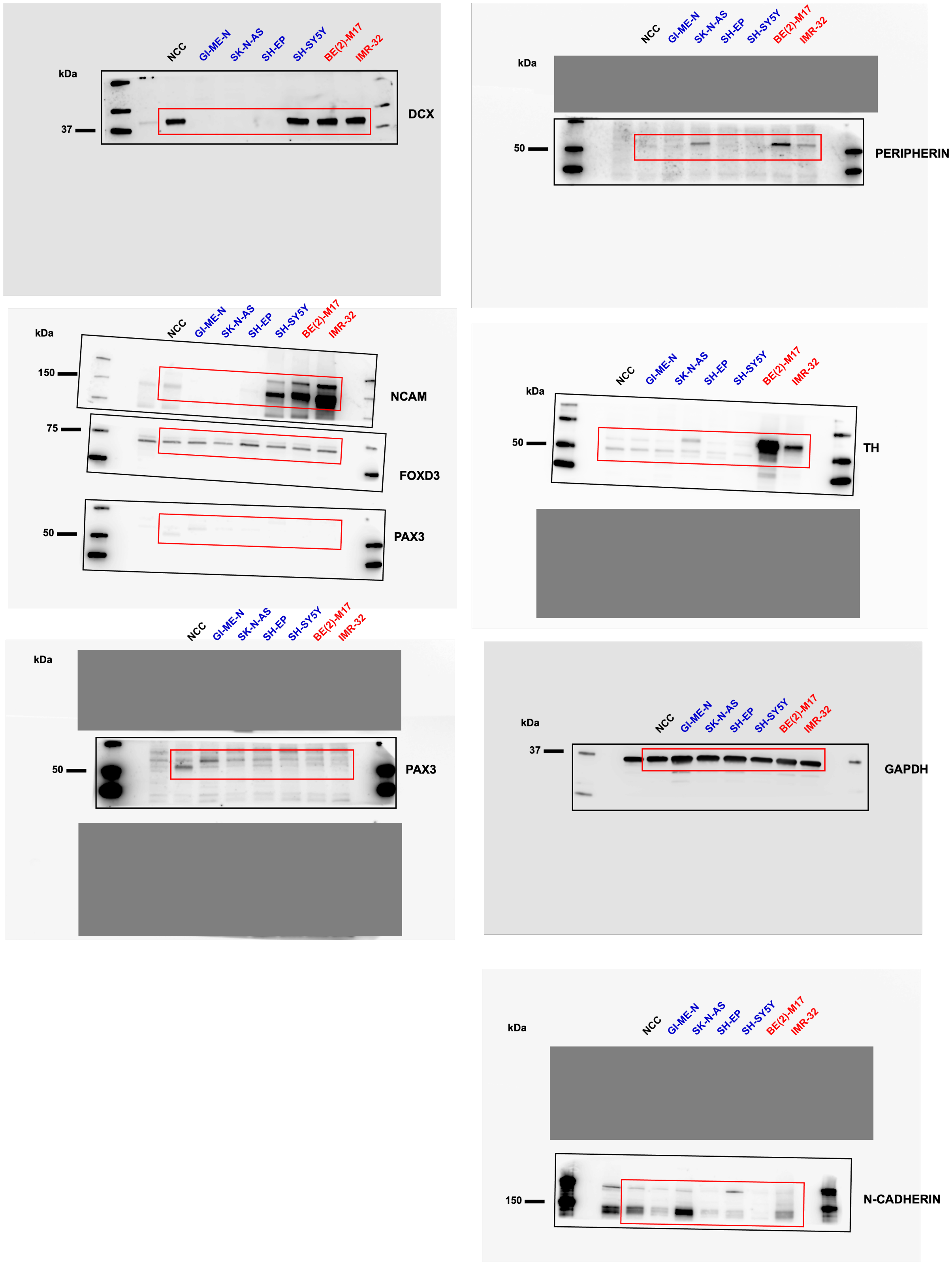

Figure 3 western blot images

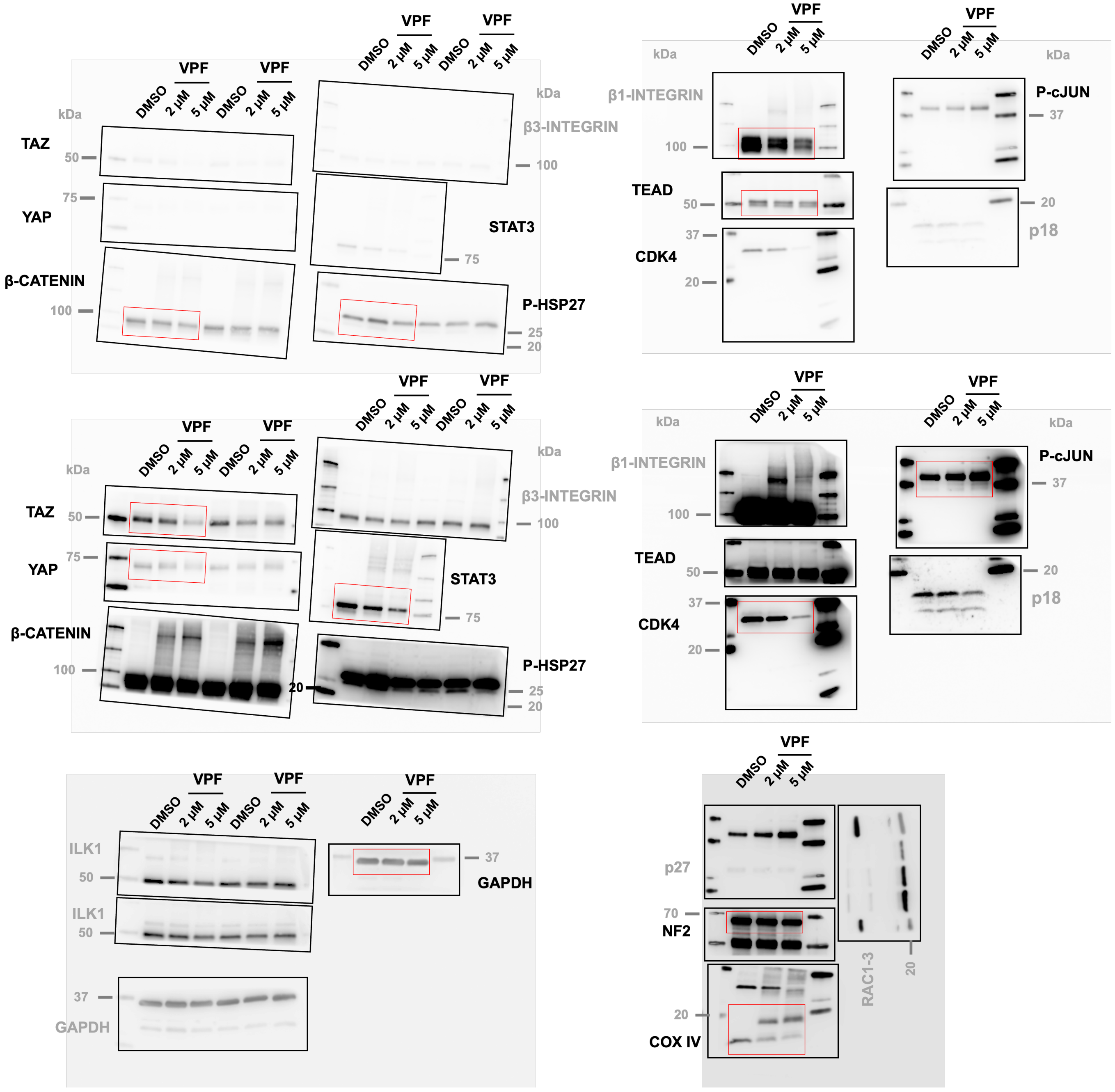

Figure 3 western blot images

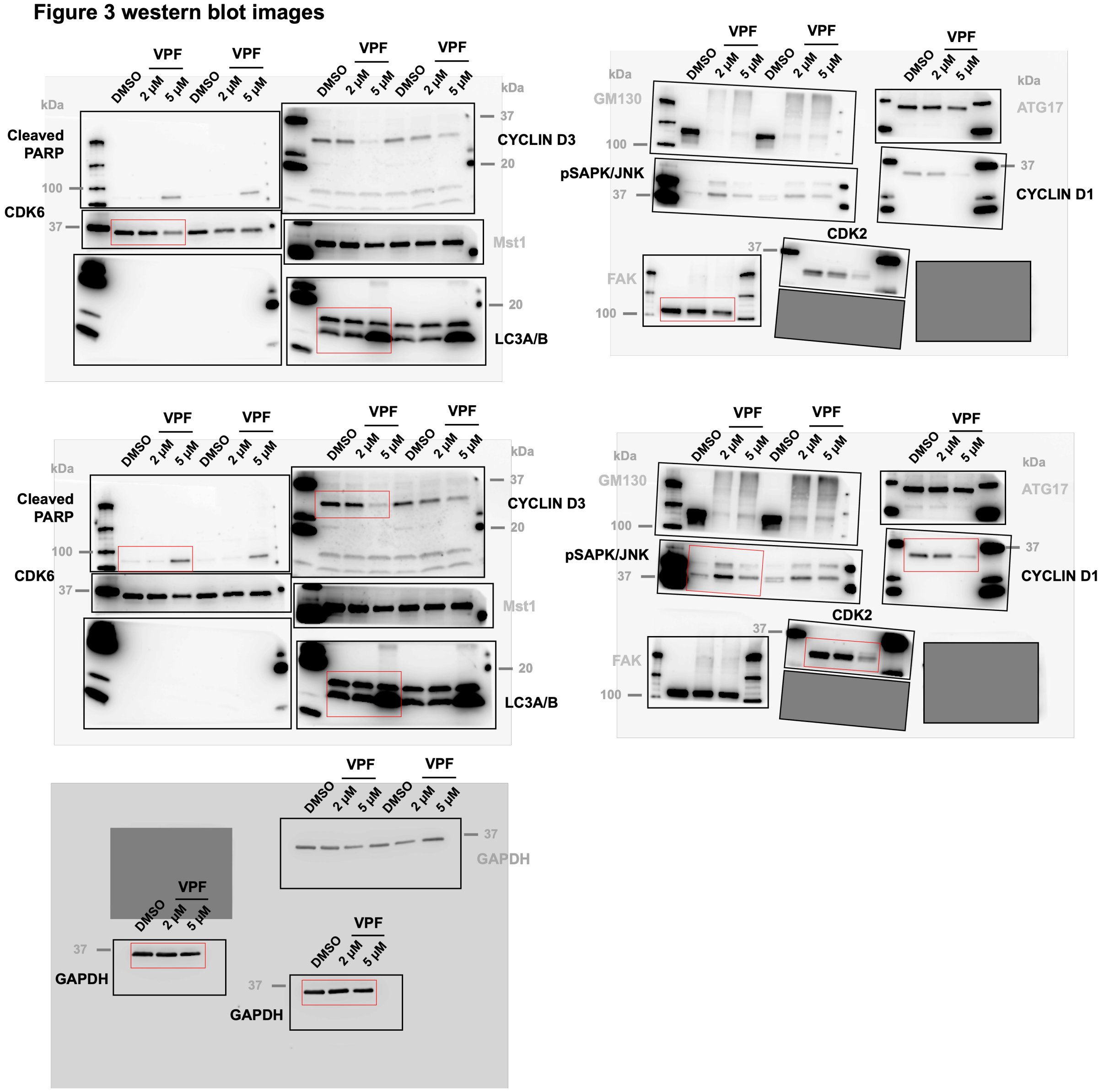

Figure 4 western blot images

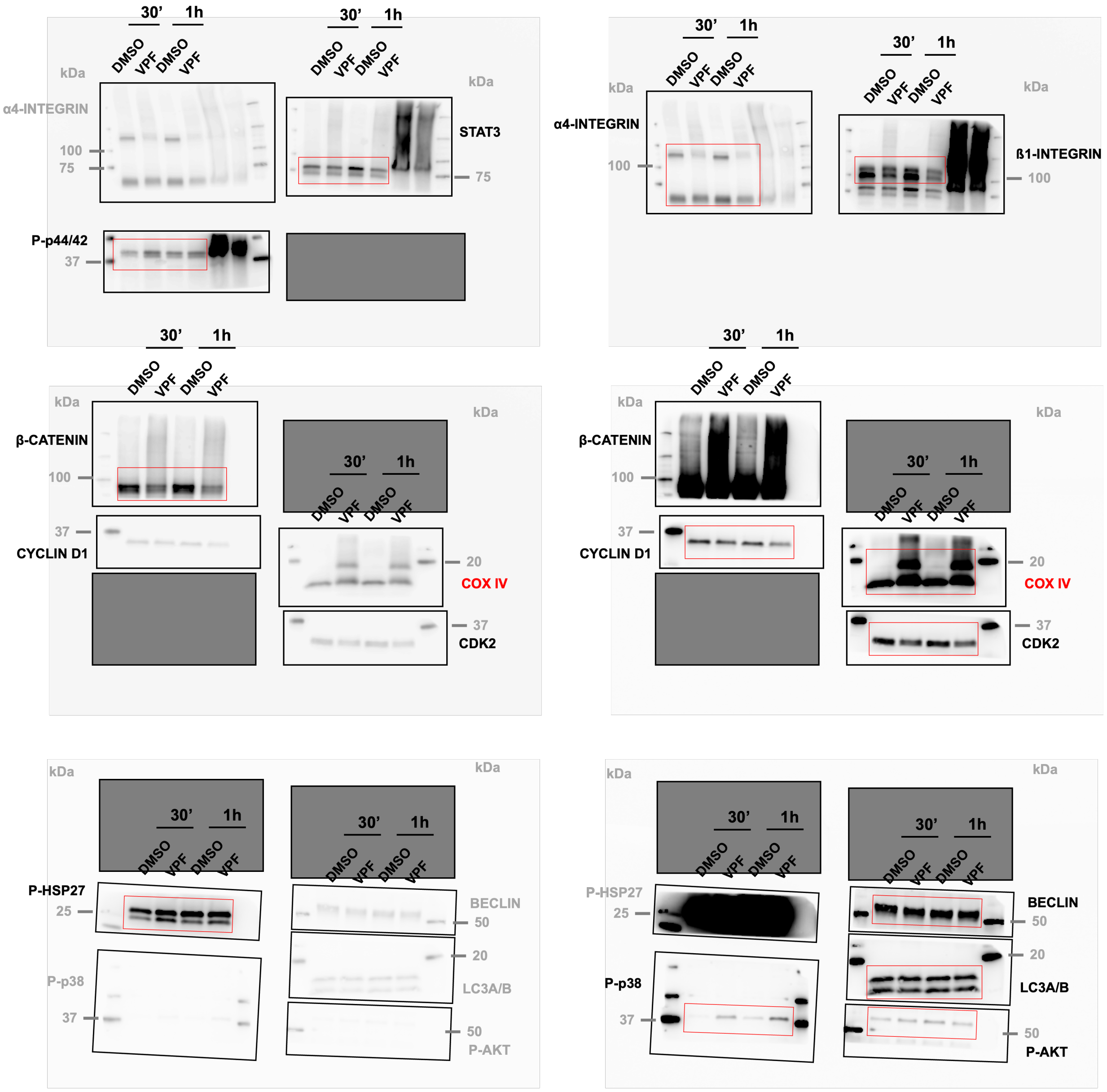

Figure 4 western blot images

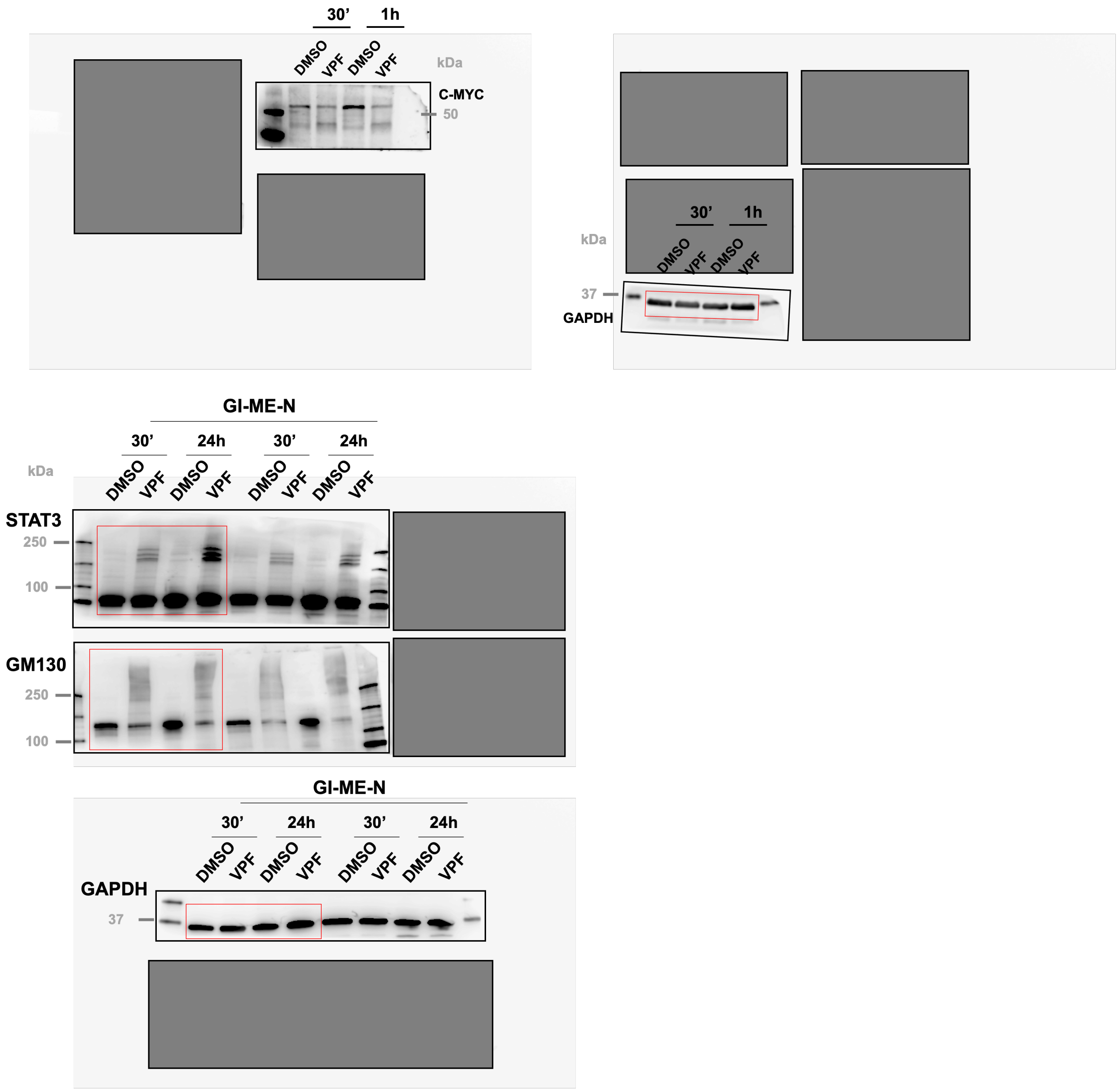

Figure 5 western blot images

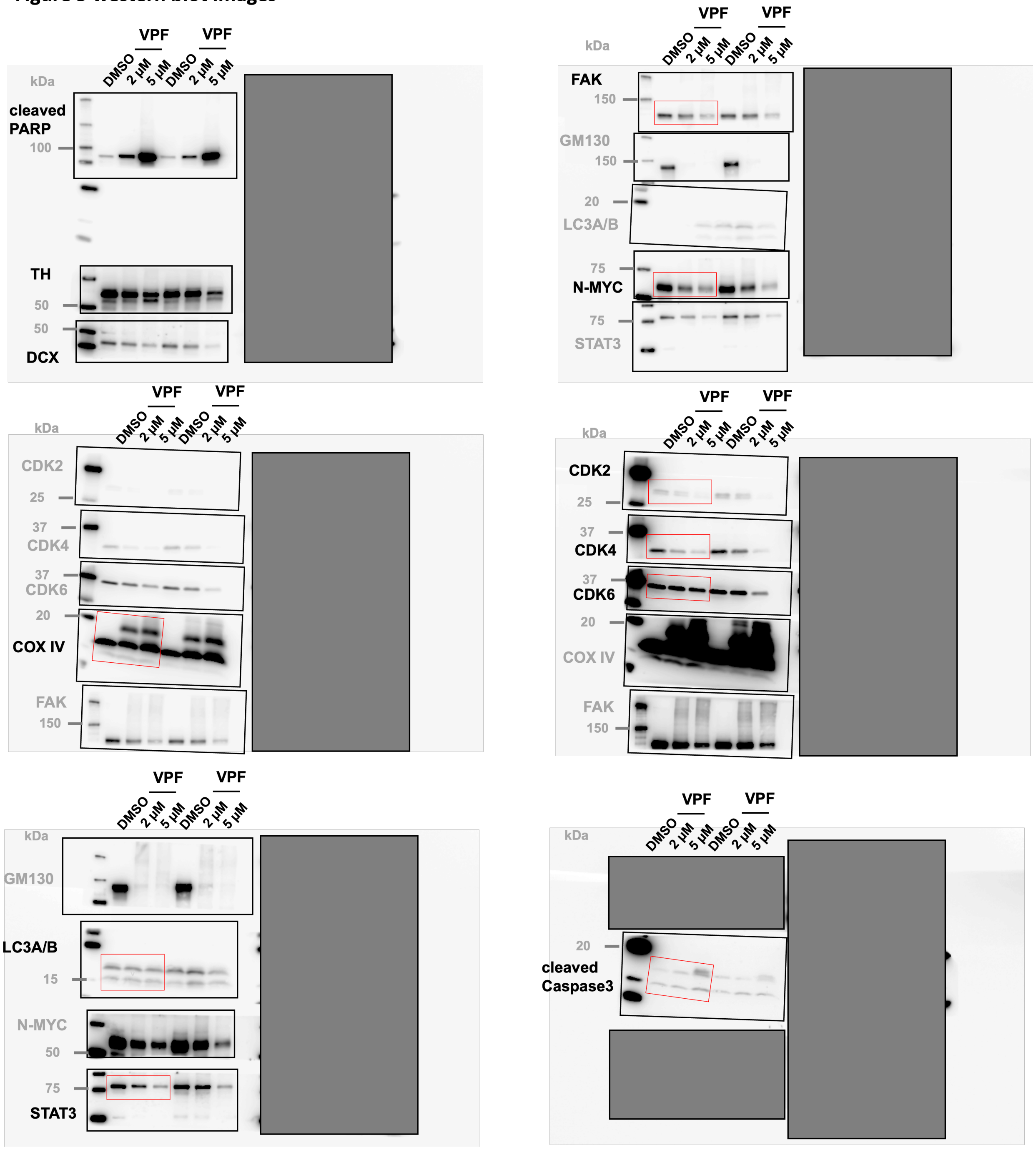

Figure 5 western blot images

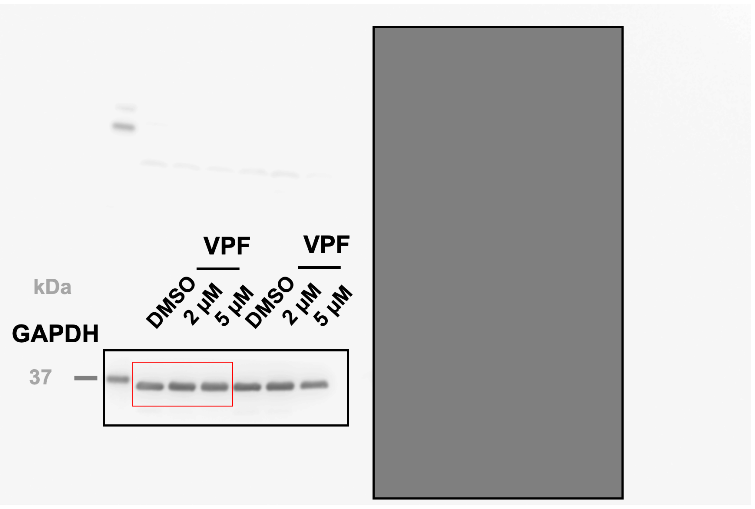

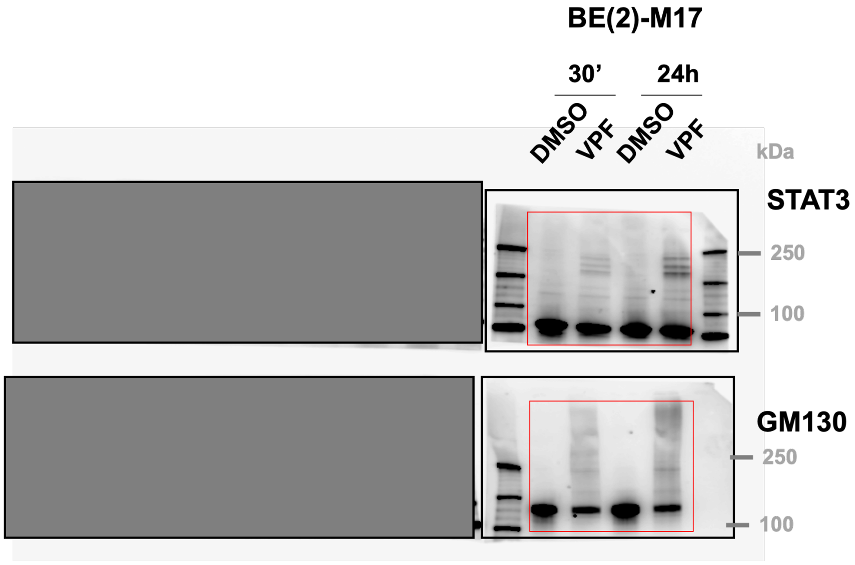

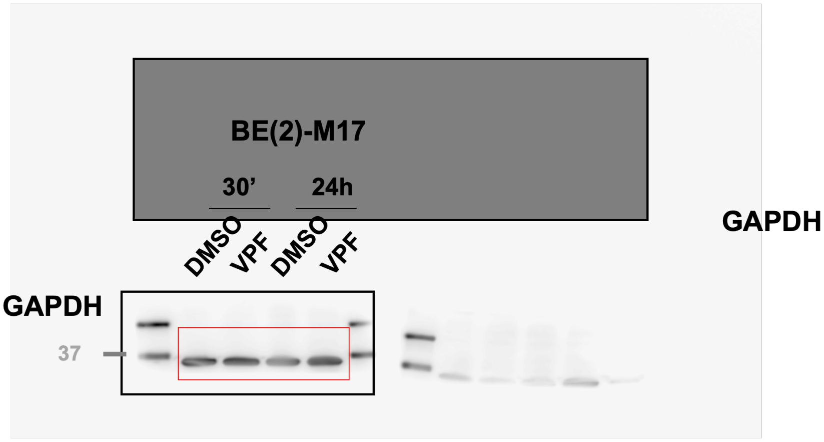

Supplementary Fig. 3 Phospho-kinase Array western blot images: GI-ME-N 24h DMSO vs 5 µM VPF

Replicate A

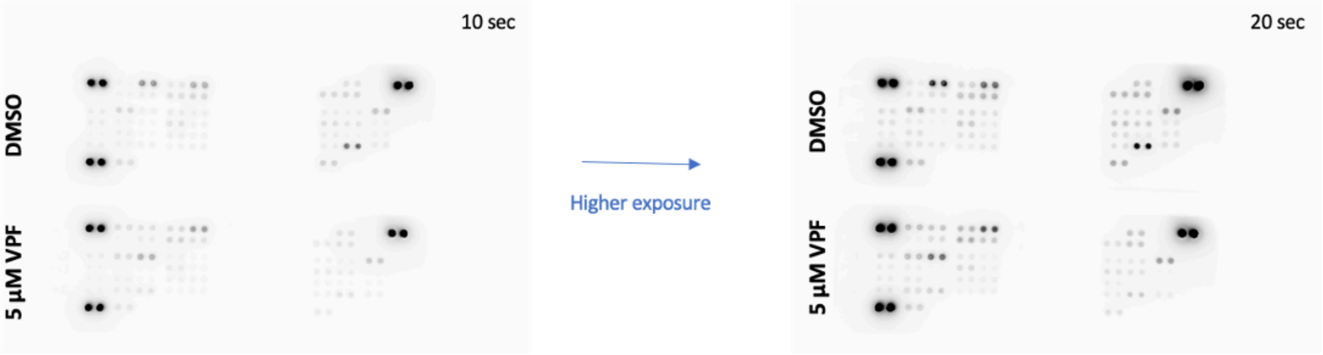

Replicate B

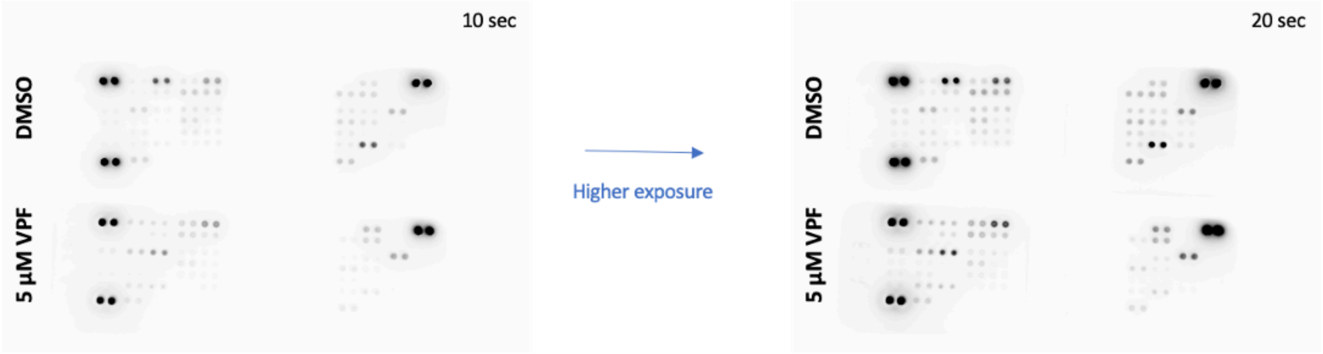

Supplementary Figure 4 western blot images

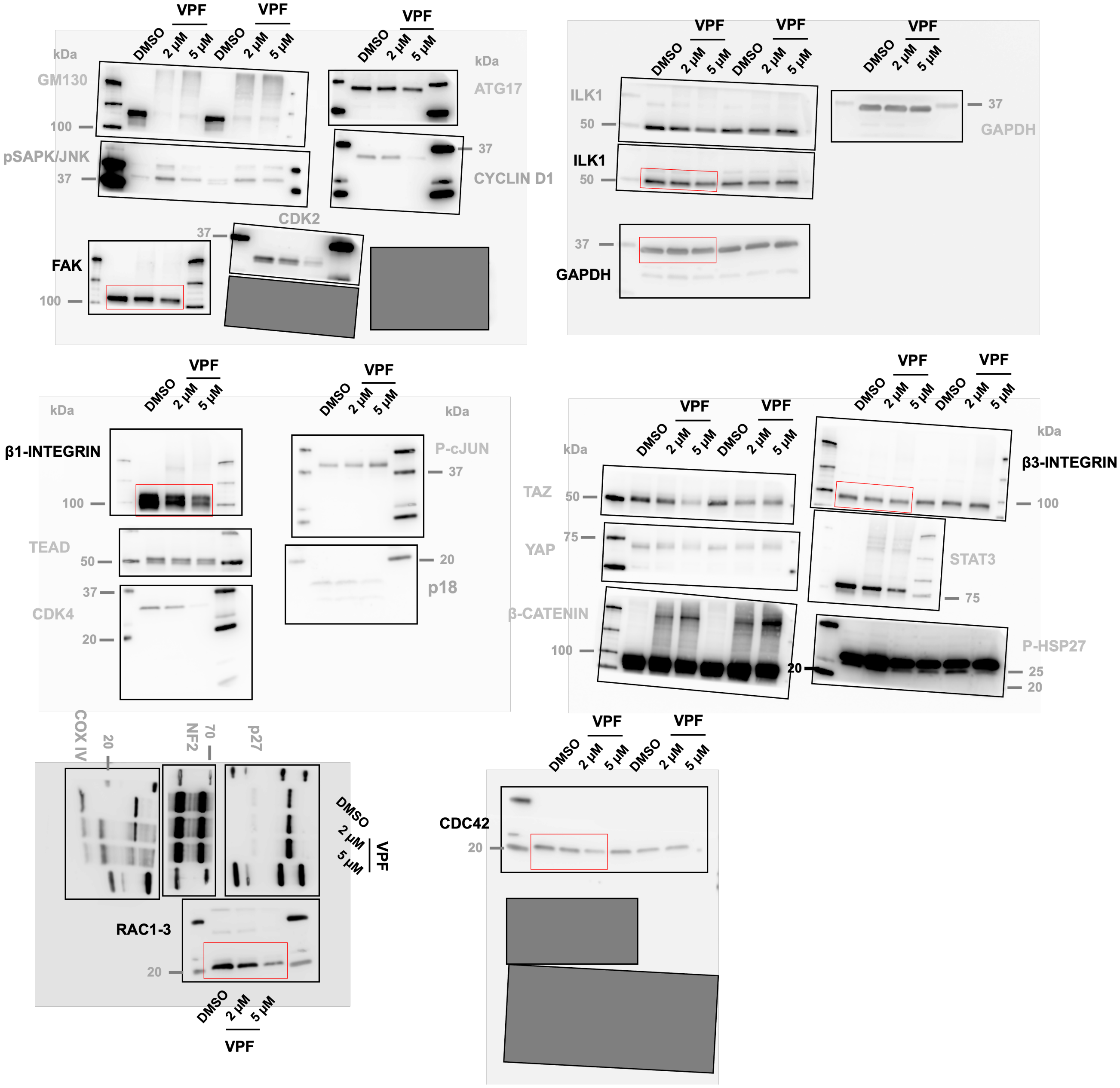

Supplementary Figure 5 western blot images

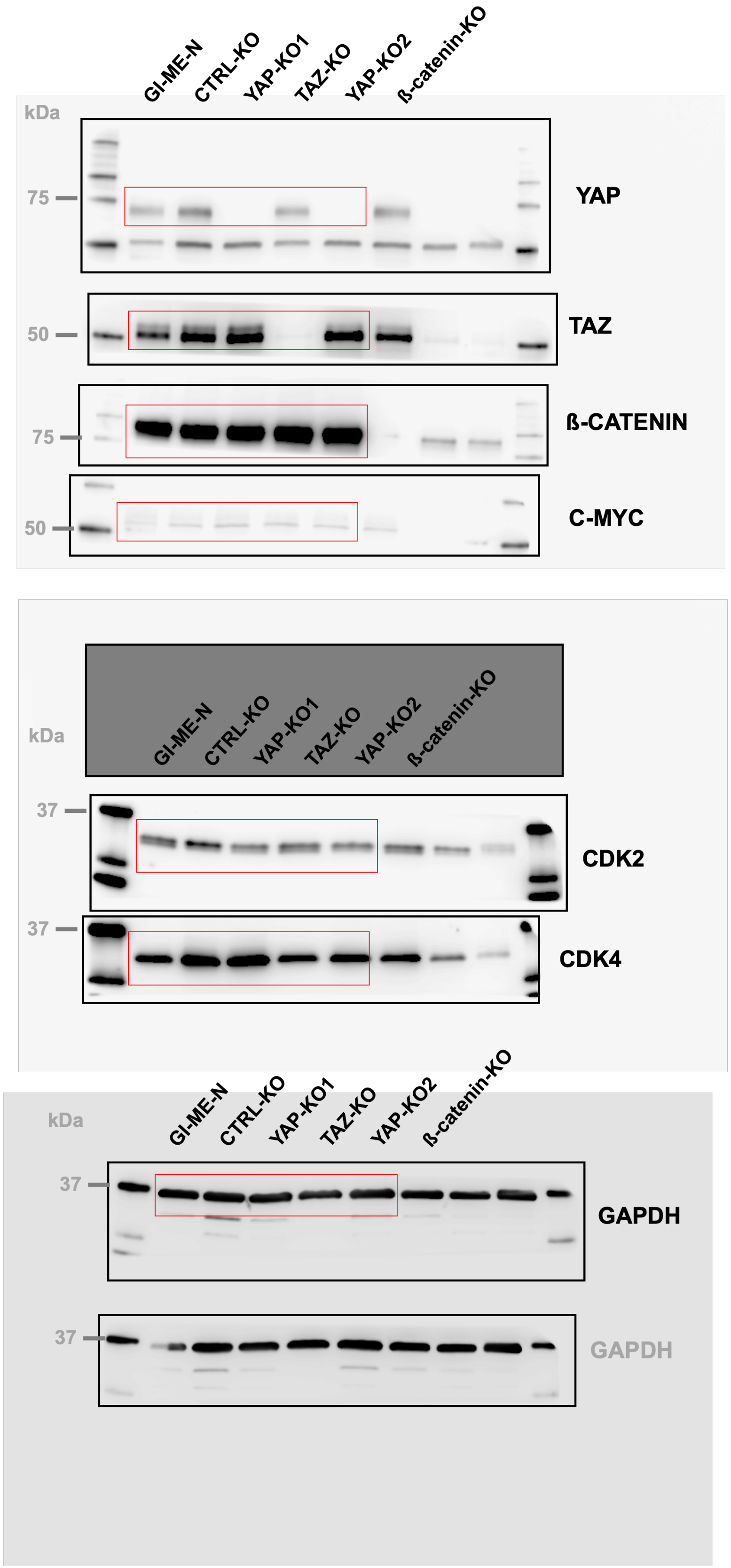

Supplement: Supplementary file 1 — Supplementary Information. [file 41598_2023_29796_MOESM1_ESM.pdf]
